# Training radial basis function networks for wind speed prediction using PSO enhanced differential search optimizer

**DOI:** 10.1371/journal.pone.0196871

**Published:** 2018-05-16

**Authors:** Hannah Jessie Rani R., Aruldoss Albert Victoire T.

**Affiliations:** Department of Electrical & Electronics Engineering, Anna University Regional Campus, Coimbatore, Tamil Nadu, India; University of Vermont, UNITED STATES

## Abstract

This paper presents an integrated hybrid optimization algorithm for training the radial basis function neural network (RBF NN). Training of neural networks is still a challenging exercise in machine learning domain. Traditional training algorithms in general suffer and trap in local optima and lead to premature convergence, which makes them ineffective when applied for datasets with diverse features. Training algorithms based on evolutionary computations are becoming popular due to their robust nature in overcoming the drawbacks of the traditional algorithms. Accordingly, this paper proposes a hybrid training procedure with differential search (DS) algorithm functionally integrated with the particle swarm optimization (PSO). To surmount the local trapping of the search procedure, a new population initialization scheme is proposed using Logistic chaotic sequence, which enhances the population diversity and aid the search capability. To demonstrate the effectiveness of the proposed RBF hybrid training algorithm, experimental analysis on publicly available 7 benchmark datasets are performed. Subsequently, experiments were conducted on a practical application case for wind speed prediction to expound the superiority of the proposed RBF training algorithm in terms of prediction accuracy.

## Introduction

Artificial neural networks (ANN) are a section of artificial intelligence systems fundamentally designed to overcome some of the challenges the mathematical models fail with complex and ill-defined problems. They are fault tolerant and solve the problem by learning from similar examples. ANN are capable of handling noisy and ambiguous data, with the ability to predict and generalize once efficiently trained [1].

Radial basis function (RBF) networks are another class of ANN simulating the locally tuned response observed in biologic neurons [2]. The structure of RBF consists of three layers, namely the input, hidden and output layers. The RBF training involves two stages, with centres of the hidden layer are determined first in a self-organising manner [3] and secondly, the weights connecting the hidden layer to the output layer are computed. Generally, RBF training is accomplished by computing the weights and biases to obtain the target output by minimizing the error function. To accomplish this, the following methods are widely used in the literature, matrix inversion techniques, gradient-based training approaches and evolutionary computation methods [4].

Thus in this section, a review of literature in the topic of various RBF training methods will be discussed. As the training phase determines the success of any network, the training of radial basis network(RBF) involves three step learning [5] which is the fastest as the centres are determined by unsupervised method and output weights are also determined by less complex algorithms. Though gradient descent method offers precise results as it involves derivatives which affects the computation time it is not preferred to use alone.

Training the neural networks by heuristic search algorithms like differential evolution (DE) [6] was previously utilized and the results are compared with gradient descent methods, however it is observed that no significant improvement in the performance because of DE. Similarly, in [7] the authors suggests solutions for stagnation of Differential evolution (DE) when used with neural networks, as the individual does not improves even under favourable conditions. Taking care of the initialization, merging DE with specified mutation operators, size of population (DE) are some of the key areas have been discussed.

In [8], as the training of both MLP and RBFN is difficult, evolutionary algorithms like Genetic Algorithm optimizes the subset of input data for determining the number of centres which helps us in elevating the over-fitting problems. In another work [9], the authors carried out short term wind speed prediction with inputs from five different meteorological stations and tested with ANN trained by PSO.

Similar works which involves neural network and PSO are experimented in [10] in order to improve the reliability of electric power generation, wind power is predicted with enhanced particle swarm optimization (EPSO) in combination with standard neural networks and the weights of the networks are optimized. For inputs like time series data in [11], GA is used to optimize all the three parameters of RBFN. Similar time series data in [12], nonlinear time varying evolution PSO was proposed for training RBFN and tuning the acceleration coefficients for short term electric power prediction in Taiwan.

Likewise, in [13] to improve the forecasting accuracy of Back propagation network (BPNN), adaptive differential evolution (ADE) is hybridized. Similarly, in [14] both the global search and local search capabilities of Adaptive PSO and BP is been efficiently exploited for finding the global optimum in the given search space. In another work [15] the authors proposed an improved dynamic PSO, together with Ada Boost algorithm, authors adjust the parameters (centers, widths, shape parameters and connection weights) to train the RBF NN.

Similar recently developed hybrid models[16]such as biogeography based optimization (BBO) algorithm is used for training Multi- layer Perceptron (MLP) networks and tested with several classification, approximation datasets. In another work [17] modified bat algorithm is employed to optimize the weights, biases and the structure of neural network and tested on classification, benchmark time series and real time series (e.g. rainfall) datasets. Again in [18], to improve the diversity of population, two strategies are proposed in modified bat inspired algorithm, proposing Ring and Master slave methods, the weights and the structure of ANN are simultaneously optimized.

To improve the performance of training the RBFN, the combination of PSO, K-NN and OSD is presented in [19]. PSO replaces K-means clustering for finding centres, as in K-means random selection of centres was deficient. Subsequently PSO [20] is used for parallel optimizing of parameters of RBFN as it handles two different swarms. These two swarms exchanged the information of optimized parameters among themselves. Again in [21], the variant of PSO i.e PSO with mutation operation is used to train the RBF parameters like the weights and sigma of activation function.

For predicting the electric load demand, a hybrid method of PSO-GA-RBF [22] is presented. Since GA is binary coded, and PSO is real value coded, this algorithm is a mixed coded one where the network structure is been optimized by GA and the weights and basis are optimized by PSO. Similarly, a Support Vector Regression (SVR) model is hybridized with the differential empirical mode decomposition (DEMD) method and PSO-support vector machine for electric load forecasting [23]. In [24], the authors tried RBFN for solar power prediction with wind speed and two dimensional representation of solar irradiation as its inputs. Again [25] uses a hybrid PSO-GA for finding the parameters of radial basis network in rainfall prediction.

Similarly, forecasting stock indices using an artificial fish swarm algorithm (AFSA) optimizes RBF is discussed in [26]. K-means clustering which is adapted for finding centres of RBF, weights linking the output and hidden layer are being optimized by AFSA. Meanwhile in [27], a hybrid perturbation artificial bee colony trainer for a local linear RBF NN is presented. Another hybrid method integrating empirical mode decomposition with adaptive neural network based fuzzy inference system (ANFIS) for short-term wind speed forecasting is presented in [28].

To improve the diversity of individuals results in higher chance to search in the direction of global optimal [29], proposes an integrated hybrid method with PSO and GA for RBFNN training. Similarly [30–32], proposes a PSO based training for RBF NN for diverse applications. [33] presents a spatial correlation model algorithm for training ANN for wind speed and power forecasting.

Generally, inconsistency of a single technique could be resolved by combining two or more techniques to overcome the deficiencies of single models and yield more accurate results [34–36]. Accordingly this paper proposes a hybrid model combining the salient features of PSO with the differential search algorithm. Thus a new hybrid optimizer called PSODS will be the trainer for the RBF NN for wind speed prediction. Before establishing the applicability of the proposed technique to train RBF NN for wind speed prediction, seven publicly available test datasets are experimented to demonstrate the results produced by the new scheme is evidently superior in many aspects compared to other reported methods for training RBF NN.

Despite the fact that any developed technique can be experimented and proved to be effective for standard test problems, it is more insistence to justify its performance on a real time system. Accordingly this research after establishing the performance of the proposed trainer for RBF NN, will be experimented on a practical wind prediction problem. Wind is one of the green renewable energy, widely available for electric power generation. In spite of its chaotic nature the wind is effectively utilized for power generation with suitable planning. Several factors influence the speed of the wind and hence prediction of wind speed will help electric power companies to well utilize the energy tapped from wind and minimize the expenses for fossil power generation.

The literature for application of NN for wind speed prediction is comprehensive. Here selective articles are reviewed pertaining to the content of this research. Wind speed prediction is done using three NN, namely adaptive linear element, back propagation and radial basis function and demonstrated that no particular NN outperforms the other in terms of all evaluation metrics [37]. In [38], a self-organising map is used to process the uncertainties of wind nature and then processed using RBF NN. Similarly an adaptive neuro fuzzy system is proposed along with similar day method and proved to be effective [39].

In [40], a NN model for predicting real time information obtained from various locations in the mountainous regions of Himalaya is presented. Similarly, a recurrent NN model is developed for predicting the wind power generated from wind turbines installed across the coastal region [41]. In another work [42], a Least square support vector machine (LSSVM), with empirical wavelet transform as a pre-processor is presented. Similarly, two different statistical models with same datasets of inputs ranges from atmospheric variables is presented [43].

Complexity is one of the key factors which trigger the advent of new solution techniques for finding possible solutions, where existing mathematical programming techniques fail. Evolutionary computation algorithms are promising alternatives, when attempting complex search space and further expounded to overcome several drawbacks the mathematical programming techniques face when applied.

The search range of a neural network, where weight determination is the key problem is also complex and cumbersome in nature. This solution space is not only a challenge for any method to produce quality solutions, there are other issues like local trapping and premature convergence. An inherent feature of most of the population based algorithm is their capability of balancing between exploration and exploitation when searching the complex solution space. Similar, the key concern and shortcoming of any evolutionary computation algorithms is to overcome the trapping into local optima and to avoid poor convergence.

Based on this three contributions are made as follows,

- A new population initialization algorithm is proposed using a chaotic sequence called ‘Logistic iterator’ ensuring the search space information can be extracted with enhancement in population diversity. In addition, an opposition based population is subsequently generated using the population generated by the chaotic sequence, to further diversify the initialization population.
- A new optimizer using the differential search algorithm is proposed with functionally modified by incorporating the local search feature of the PSO. Thereby the exploration of DS is ensured and exploitation of the PSO is well utilized.
- The newly proposed optimizer named as PSO enhanced differential search (PSODS) algorithm will be used to train the radial basis function neural networks and the best possible settings for centroid, spread and weights will be estimated and demonstrated for its suitability in solving both theoretical and practical applications of prediction.

The rest of this paper is organized as follows. Section 3, presents a brief introduction to the RBF NN followed by the Logistic chaotic sequence based Initial population generation algorithm. Subsequently with the brief overview of DS algorithm and PSO algorithm, the modeling of the PSODS algorithm for training the RBF NN is presented in Section 4. Section 5, summarizes the simulation results of the seven publicly available regression test datasets and finally for wind speed prediction problem. Finally the paper concludes by summarizing the merits of the proposed approach.

## List of symbols

*w*_*jk*_ Weights of hidden layer *k* linked with *j* output layer.
*μ*_*k*_ Centroids of hidden layer neuron *k* ofRBF NN.
*σ*_*k*_ Spread of hidden layer Radial basis function *k*.
*δ*_*k*_ Activation functions of hidden layer neuron *k*.
*ψ*_*ki*_ Gaussian activation function of hidden neuron *k* ofall inputs *i*
POP Population Size
DIM Dimension
MUT Mutation strategy
DNR Donor or the target of superorganism.
*chrnd*_*k*,*j*_ Chaotic random generation for *k* iteration of *j*dimension.
*oppx*_*i*,*j*_ Opposition based learning of ipopulation of *j*dimension.
$v_{id}^{t},v_{i1}^{t-1}$ Present and Previous velocities of the particle
$P_{id}^{t}$ Local best value of the particle
$x_{id}^{t}$ Present position of the particle
$p_{gd}^{t}$ Global best position of the particle
*spo* Super organism population
*ρ*_*gd*_ Gamma distribution based random number generation.
$cv_{i}^{t}$ velocity updation of each particle in the super organism.
$cspov_{i}^{t+1}$ New position of superorganism after position updation.
$spo_{i}^{t}$ Current Position of super organisms

## Radial basis function neural network (RBF NN): An overview

**Fig 1:**
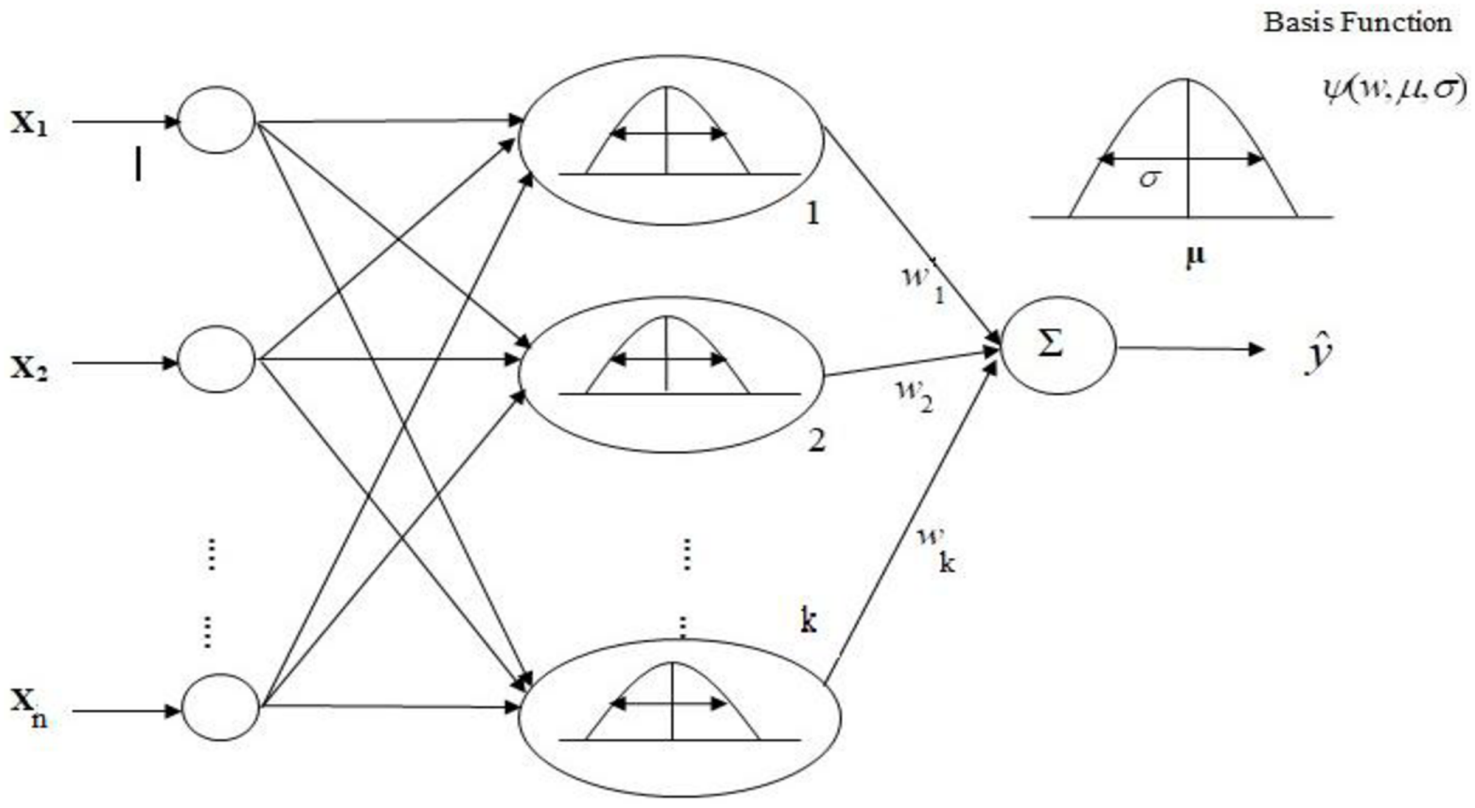
Schematic diagram of RBFNN architecture.

Radial basis function neural network RBF NN [2,3] is the general class of non-linear and three-layer feed forward neural networks: (i) an input layer with *n* nodes, (ii) a hidden layer with *m* neurons or RBFs, and (iii) an output layer with one or several nodes (Fig 1). The unsupervised layer is defined between input nodes and the hidden neurons in the RBF network, while the supervised layer exists between hidden neurons and the output nodes.

The *j*^*th*^ output *y*_*j*_*(i)* in the network can be defined as

$$y_{j}(i)=\sum_{k=1}^{m}w_{jk}\delta_{k}\left[x(i),\mu_{k},\sigma_{k}\right],j=1,2,\ldots \ldots ‥,n,i=1,2,\ldots \ldots ‥n,$$ (1)

Where ‘k’ is the number of used RBFs, *w*_*jk*_ (*k* = 1,2,………,*m*) is the weights of RBF network linked with *j*^*th*^ output, the centroid (center) vector *μ*_*k*_ = [*μ*_1_, *μ*_2_,…..*μ*_*m*_]^*T*^, and the spread vector of RBF NN *σ*_*k*_ = [*σ*_1_, *σ*_2_,…..*σ*_*m*_]^*T*^ The spread is generally calculated as,

$$\sigma_{k}=\frac{Maximum\;dis\text{tan}ce\;between\;any\;2\;centers}{\sqrt{number\;of\;centers}}=\frac{d_{\text{max}}}{\sqrt{m_{1}}}$$

The weights connecting hidden layer to output layer is given by, *w*_*k*_ = [*w*_1_, *w*_2_,….., *w*_*m*_]^*T*^

According to [30], the basis function can be defined in several ways, while some of the most commonly used basis functions are as follows: Gaussian, multi-quadric, inverse multi-quadric, generalised inverse multi-quadric, thin plate spline, cubic and linear function. In this study, the RBF is represented by the Gaussian function that acts as the activation function for the neurons in the hidden layer formed by every term *δ*_*k*_. The output layer applies a linear combination of this function and is represented as

$$\delta_{k}(x,\mu_{k},\sigma_{k})=\overset{n}{\underset{i=1}{∐}}\psi_{ki}(x_{i},\sigma_{ki},\mu_{ki})$$ (2)

The Gaussian form is defined as

$$\psi_{ki}(x_{i},\mu_{ki},\sigma_{ki})=e^{-\left(\frac{{\left(x_{i}-\mu_{k_{i}}\right)}^{2}}{2\sigma_{{{}_{k}}_{i}}^{2}}\right)}$$ (3)

Now, the *j*^*th*^ output becomes:

$$y_{j}(i)=\sum_{k=1}^{m}w_{jk}e^{-\sum_{i=1}^{n}\left(\frac{{\left(x_{i}-\mu_{k_{i}}\right)}^{2}}{2\sigma_{{{}_{k}}_{i}}^{2}}\right)}$$ (4)

The parameters of RBFN such as *w*_*jk*_, *μ*_*k*_, *σ*_*k*_ are to be optimised so that the error function as stated below is minimised. M is the number of sample used to train the RBF NN.

$$RMSE=\sqrt{\frac{\sum_{j=1}^{M}{\left(t_{j}-y_{j}(i)\right)}^{2}}{M}}$$ (5)

The key problem in RBF neural network structure is to judge the number of hidden layer neurons and their corresponding spread, *σ*_*k*_ and centroids *μ*_*k*_. To obtain the above parameters to design the RBF neural network used for prediction problems, the root mean square error (RMSE) is formulated as an optimization problem. Accordingly the fitness function for the optimization procedure is given below.

### Fitness function

The fitness function is the key element in determining the suitable parameters for the better performance of the RBF NN. For a dataset with samples S = {(X_j_,Y_j_), j = 1,2,3,…M}. where, X_j_ is the j^th^ sample given by X_j_ = {(x_j_), j = 1,2,….J}, where ‘M’ is the number of samples, *n* is the number of inputs. *t*_*j*_ is the output as per data and *y*_*j*_(*i*) is the output estimated by RBF NN for the input sample X_j_. Thus the error function also considered as fitness function is given by Eq (5). Thus this paper, proposes a new hybrid optimizer to determine the appropriate weights *w*_*jk*_, the spread, *σ*_*k*_ and centroids *μ*_*k*_, as they are of particular importance for the better performance of the RBF neural network.

## Logistic chaotic sequence based Initial population generation

In any evolutionary computation procedure, the convergence speed and final optimum solution obtained are greatly influenced by the initialization of the candidate solution or population. Mostly, initial candidate solutions are randomly generated within the range of the variables limits as no information of the solution space is available [44,45]. Recently, several evolutionary computation procedures due to the randomness and sensitivity dependence on the initial conditions, adopts chaotic maps for initialization of the candidate solutions as chaotic maps are capable of extracting diversity within the solution space, thereby generate initial population that are much diversified (throughout the search space) than the regular randomly initialized population.

In this work the chaotic map adopted is the one proved to be most successful in various applications. Thus in this work the Logistic iterator [46] is selected and its equation is given as follows:

$$chrnd_{k+1,j}=\alpha .chrnd_{k,j}(1-chrnd_{k,j})$$

Where, *chrnd*_0,*j*_ = 0.2027 and *α* = 4

Subsequently once the population initialization is done with chaotic maps, another improvisation is carried out by applying the opposition based population diversification [47]. This diversification is done for the entire size of the population and their place in the search will be decided based on their fitness. So that out of twice the size of the candidates only the first half candidates with highest fitness will enter the PSODS routine. The combined algorithm for population initialization using chaotic maps and opposition based method is presented in Algorithm 1.

### Algorithm 1: Chaotic opposition-based population initialization

01: The maximum number of chaotic iteration CHITR is set to 300,

02: population size is POP.

 {---Chaotic systems---}

03:**for** i = 1 to POP **do**

04: **for** j = 1 to DIM **do**

05:  Initialize the variables randomly from the limit prescribed

06:   **for** k = 1 to CHITR **do**

07: *chrnd*_k+1,j_ = *α*.*chrnd*_*k*,*j*_ (1 − *chrnd*_*k*,*j*_)

08:   **end for**

09: *x*_*i*,*j*_ = *x*_min,*j*_ + *chrnd*_*k*,*j*_ (*x*_max,*j*_ − *x*_min,*j*_)

10: **end for**

11:**end for**

 {------Opposition—based learning method--------}

13:**for** i = 2 instep of 2 to POP **do**

14: **for** j = 1 to DIM **do**

15: *oppx*_*i*,*j*_ = *x*_min,*j*_ + *x*_max,*j*_ − *x*_*i*,*j*_

16: **end for**

17: **end for**

18: The POP fittest individuals with better fitness out of 2xPOP will be the initial population.

## Differential search algorithm: An overview

The Differential search (DS) is one of the recently developed evolutionary computation procedure to solve constrained global optimization problems. It is getting attention in recent times in wide range of applications which requires rigorous search of solution space [48,49]. The DS algorithm shall be briefed in three stages as follows:

- Set of candidate solutions of a particular problem shall be considered as artificial- super-organism migrating towards better fitness.
- In the course of migration, the artificial-super-organism examines whether a randomly selected location (stop over site) is suitable for temporarily settlement.
- If the location (based on fitness evaluation) is suitable to stall over during the migration, the super-organism that made this location will position itself there.

**Fig 2:**
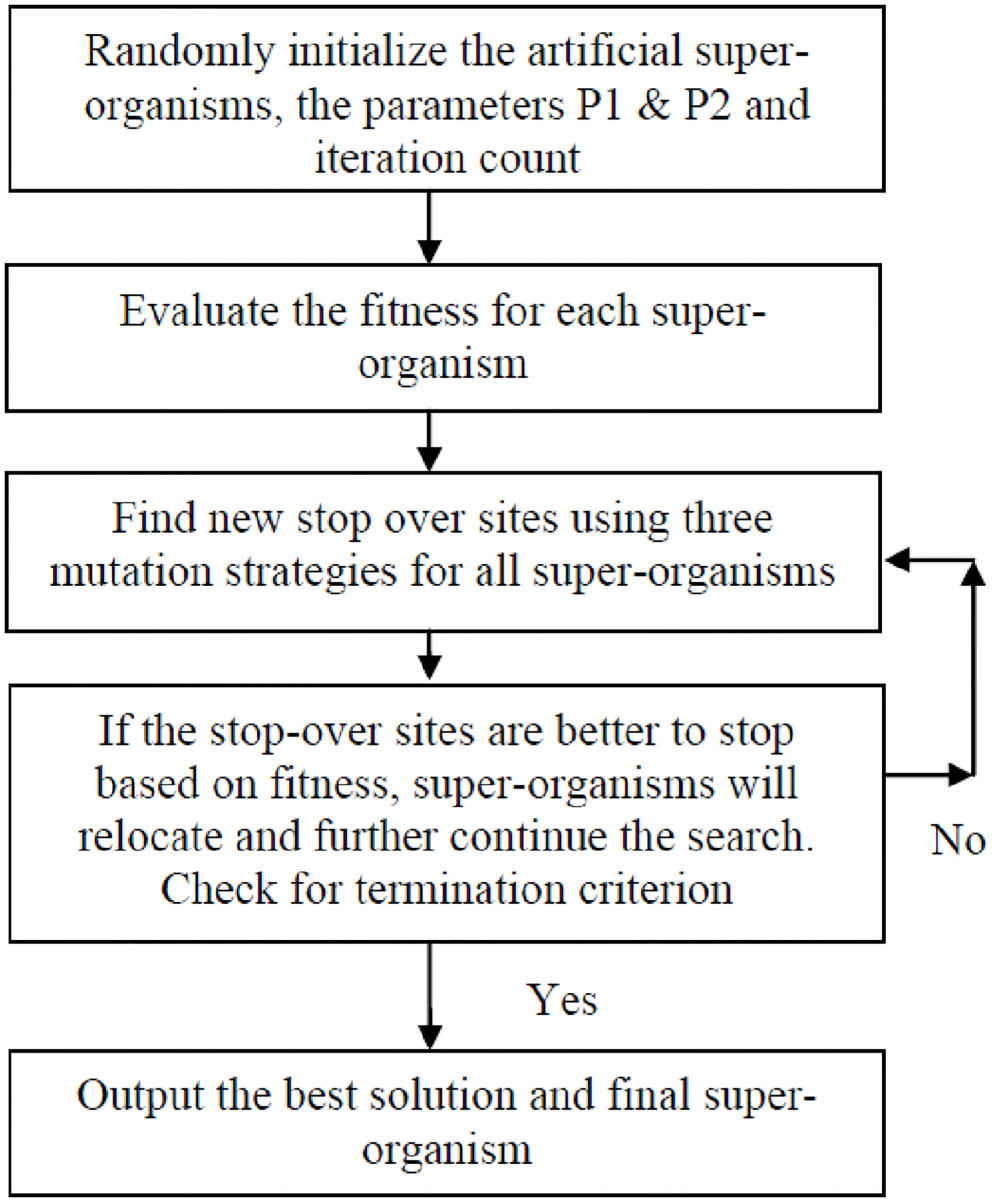
Flowchart of the differential search algorithm.

The above procedure will be continued until all the artificial-super-organism examines and settle at an acceptable position as per the problem requirement. The DS procedure is inspired by the movement of a super-organism well similar to the Brownian-like random-walk model. The flow chart of the Differential search algorithm is shown in Fig 2.

The salient feature of the DS Algorithm is, only two parameters (P1 and P2) are normally to be appropriately set for the algorithm to search for the better solution. DS is very simple with good exploration capability but poor at exploitation. Hence requires large number of iterations to obtain good result.

## Particle swarm optimization: An overview

Introduced as a simple real number optimization algorithm, PSO is the widely used swarm intelligence algorithm in variety of applications [34,35]. The algorithm is inspired from the behavior of bird flocks known as a swarm in search of food. It’s simple steps in reaching a quality solution with control over both global and local search capability made it a popular optimization algorithm. The PSO algorithm shall be briefed in three stages as follows:

- Set of candidate solutions of a particular problem shall be considered as particles with positions in the search space moving towards better fitness
- The movement of particles will be based on their own personal information and all other particles information in the search space.
- If there is a better fitness found during the movement, the particles will move to the new position or else stay where they are.

**Fig 3:**
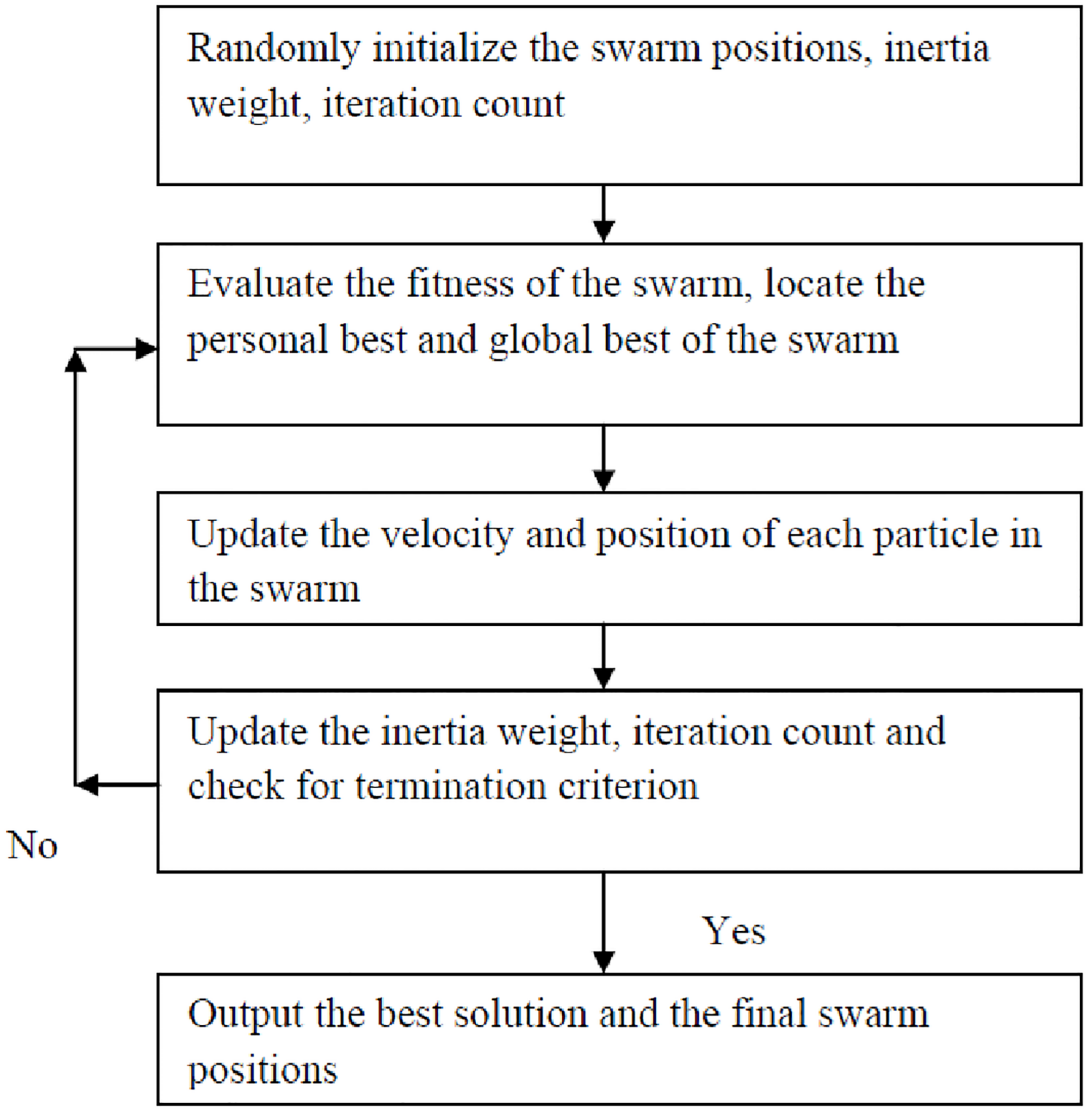
Flowchart of the particle swarm optimization algorithm.

The above procedure will be continued until all the particles update their positions and settle at an acceptable position as per the problem requirement. The flow chart of the PSO algorithm is shown in Fig 3.

One of the features of the PSO algorithm is its ability to have control over both global and local search of the solution space. In order to realize this, the algorithm is modelled with linearly decreasing inertia weight which at the beginning supports global exploration and at the end it ensures local exploitation. One of the significant weakness of PSO algorithm is during the exploration the particles often miss better solution region. Additionally, in PSO when the particles positions are updated neglecting the previous velocities, they tend to lead to local search of the region where the particles position.

In this research work, the PSO will use a neighbourhood topology to exploit the solutions of DS by thorough search of solution region. This neighbourhood topology will be based on ring topology with neighbours fetched considering both fitness and candidates themselves. Accordingly the velocity equation given in Eq (6), will be modified as in Eq (7).

$$v_{id}^{t}=v_{i1}^{t-1}+c_{1}r_{1}(P_{id}^{t}-x_{id}^{t})+c_{2}r_{2}(p_{gd}^{t}-x_{id}^{t})\;\;d=1,2,\ldots ,D$$ (6)

$$v_{id}^{t}=r_{1}(P_{id}^{t}-x_{i-1d}^{t})+r_{2}(P_{id}^{t}-x_{i+1d}^{t})\;\;d=1,2,\ldots ,D$$ (7)

Where, $v_{id}^{t},v_{i1}^{t-1}$ is the present and previous velocities of the particles, *c*_1_, *c*_2_, *r*_1_, *r*_2_ are scale and random numbers. $P_{id}^{t},\;x_{id}^{t}\&p_{gd}^{t}$ are local best, present position & global best of the particles of ‘d’ dimensions, $x_{i-1d}^{t},x_{i+1d}^{t}$ are neighbour of the particles $\;x_{id}^{t}$

In this research, a new topology will be used where both the fitness and candidates neighbours are chosen for updating the solutions. This will be explained in detail in the next section.

### PSO enhanced differential search optimizer

In this section, the new hybrid algorithm PSODS that functionally integrates PSO with DS will be discussed in detail. As discussed earlier, DS is very simple with good exploration capability but poor at exploitation. Similarly, PSO algorithm is good in exploitation with adjustment in its inertia weight. Hence taking the advantages of both the techniques, a new hybrid technique is formulated.

#### Step by step procedure of PSODS algorithm

**1**. **(Initialization and Generation of the initial artificial organism)**

Initialize the super-organism population Such that, POP is the size of super-organism, and K is the dimension of the problem also assumed as the size of one clan. In addition assume initial values for control parameters *P*_1_ & *P*_2_

Randomly initiate artificial organism using the chaotic sequence initialization procedure discussed in section 4 (Algorithm 1),

Such that, the artificial organism should describe the RBF NN with K numbers of hidden layer neurons, should comprise of the parameters *w*_*ik*_, *μ*_*k*_, *σ*_*k*_ to be minimized and expressed as

$$\begin{array}{l} t_{1}=(w_{11}^{i},w_{12}^{i},\ldots \ldots ,w_{1K}^{i},\sigma_{11}^{i},\sigma_{12}^{i},\ldots ‥,\sigma_{1K}^{i},\mu_{11}^{i},\mu_{12}^{i},\ldots .,\mu_{1K}^{i})\\ t_{2}=(w_{21}^{i},w_{22}^{i},\ldots \ldots ,w_{2K}^{i},\sigma_{21}^{i},\sigma_{22}^{i},\ldots ‥,\sigma_{2K}^{i},\mu_{21}^{i},\mu_{22}^{i},\ldots .,\mu_{2K}^{i})\\ -\\ -\\ -\\ t_{M}=(w_{M1}^{i},w_{M2}^{i},\ldots \ldots ,w_{MK}^{i},\sigma_{M1}^{i},\sigma_{M2}^{i},\ldots ‥,\sigma_{MK}^{i},\mu_{M1}^{i},\mu_{M2}^{i},\ldots .,\mu_{MK}^{i}) \end{array}$$

Thus, each artificial organism is functionally expressed as *T*_*p*_

*T*_*p*_ = {*t*_1_, *t*_2_, *t*_3_,…., *t*_*M*_}, *p* = 1,2,3, …., *POP*, M is the number of samples used for training.

The randomization is done in the range of [-1,+1] for *w*_*ik*_, *σ*_*k*_[-,1+1] and [0,1]for *μ*_*k*_

**2**. **(Fitness Function Evaluations)**

Each artificial organism should be evaluated for its fitness value using the fitness function designed for the problem of interest.

Thus the fitness function here is $fit=RMSE=\sqrt{\frac{\sum_{j=1}^{M}{\left(t_{j}-y_{j}(i)\right)}^{2}}{M}}$

The super-organism population is Initialized as *spo* = *T*_*p*_ and *FT*_*spo*_ = *fit*

3. Start PSODS, Iteration starts, IT = 1

**{--- Phase 1**—**Differential search algorithm ---}**

4. Locating the stop-over site for each artificial organism

This stage involves three sub-stages, they are

***Determination of Scale Factor*** ***(η)***

Scale factor is determined using,

$$\eta =\rho_{gd}[2*\rho_{1}]*(\rho_{2}-\rho_{3})$$

Where, *ρ*_*gd*_ generates random numbers from gamma distribution and *ρ*_*i*_ ~ *U*(0,1) random numbers generated.

***Determination of Donor organisms*** ***(DNR)***

The Donor or the target is determined by shuffling the super-organism and is expressed by,

DNR=RND.PERM(spo)

***Estimation of new temporary position*** ***(MUT)***

In this stage three mutation strategies are adapted, the control parameters are assumed as,

 *P*_1_ = 0.3*ρ_4_, *P*_2_ = 0.3*ρ_5_. In addition *ρ*_*i*_ ~ *U*(0,1),*i* = 1,2,3,…10

Mutation strategy in three parts as follows,

{---*Strategy part 1*---}

 *If*, *ρ*_6_ < *ρ*_7_ then,

 *If*, *ρ*_8_ < *P*_1_,

 *MUT* = *Rand*(POP,K)

  *for* it1 = 1: POP

  map(it1,:) = map (it1,:)<*ρ*_9_

 end *for*

 else*if*

{---*Strategy part 2*---}

 *MUT* = *ONES*(POP,K)

 *for* it2 = 1: POP

  *MUT*(it2, randi (size of K)) = map(it2, randi (size of K))<*ρ*_10_;

 end *for*

end*if*

else

{---*Strategy part 3*---}

*MUT* = *ONES*(POP,K)

*for* it3 = 1: pop

g = randi (K,1,(P_2_x K))

  *for* it4 = 1: size(g)

   *MUT* (it3,d(it4)) = 0

  end *for*

 end *for*

end*if*

Based on the above three mutation strategies, the stop over-sites of the super-organisms are calculated using

$$spo_{NEW}=(spo)+(\eta .*MUT).*(DNR-spo)$$

$$spo_{OLD}=spo$$

Evaluate fitness values for the new stopover site and call it as $FT_{spo}^{New}$ The new and old super-organisms are together sorted based on their fitness $FT_{fnl}=[FT_{spo}FT_{spo}^{New}]$ and the first ‘POP’ super-organisms will be used by PSO routine to further improvisation of the solution.

**{--- Phase 2**—**PSO algorithm for exploitation ---}**

PS01: 01: Select the size of the swarm *n*_*ps*_ (here 10% of super-organisms) with higher fitness value, set iteration count ‘t_max_’

PS02: Start the iteration t = 1

PS03: Choose $spo_{i}^{t}\;,\;i\in (1,2\ldots n_{ps})$ to update the velocity and thereby position of each particle,

Generate $l_{p}\sim U(0,1),\;p=1,2,3$

$$cv_{i}^{t}=l_{1}(spo_{i}^{t}-spo_{i-1}^{t})+l_{2}(spo_{i}^{t}-spo_{i+1}^{t})$$

$$cspo_{i}^{t+1}=cv_{i}^{t-1}+spo_{i}^{t}$$

Evaluate the fitness using newly generated position,$FT_{cspo}=fit(cspo_{i}^{t+1})$

$$fv_{i}^{t}=l_{1}(spo_{i}^{t}-spo_{fit,i-1}^{t})+l_{3}(spo_{i}^{t}-spo_{fit,i+1}^{t})$$

$$fspo_{i}^{t+1}=fv_{i}^{t-1}+spo_{i}^{t}$$

Evaluate the fitness using newly generated position,$FT_{fspo}=fit(fspo_{i}^{t+1})$

PS04: Compare the fitness *FT*_*fspo*_, & *FT*_*cspo*_ with fitness of the $spo_{i}^{t}$, improvement in fitness value will replace the position of $spo_{i}^{t}$.

PS05: Check for t = = t_max_, Else t = t+1, Repeat from PS03

END PSO routine

5. Check for IT = = IT_MAX_

END PSODS

**Fig 4:**
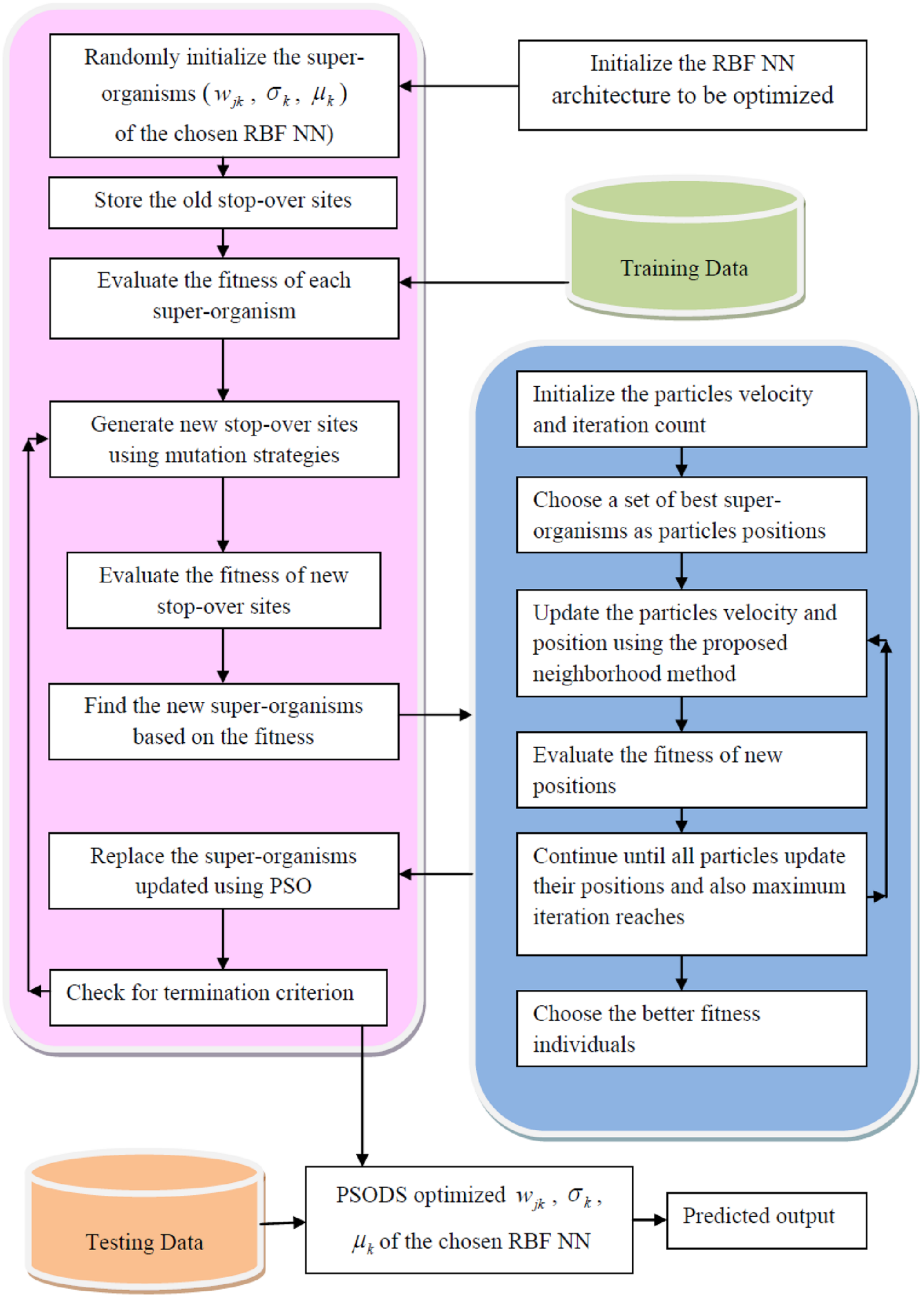
Flowchart of the proposed PSODS algorithm.

A flowchart of the PSODS algorithm is shown in Fig 4 for easy understanding. In the next section detailed experiments are performed to demonstrate the applicability of the proposed algorithm to train RBF NN and predict regression samples.

The proposed methodology was experimented on seven publicly available benchmark datasets available with the UCI Machine Learning Repository [50–52]. The seven datasets are Boston housing, Concrete Compressive strength, Airfoil self -noise, Istanbul Stock Exchange, Forest Fires, Abalone and Auto MPG. Table 1, summarizes the Benchmark Dataset Description.

**Table 1:** Description of public datasets.

Datasets	Attributes	Training data	Testing data	No. of Samples
**Boston housing**	13	253	253	506
**Concrete Compressive strength**	8	680	350	1030
**Airfoil self -noise**	5	1000	503	1503
**Istanbul Stock Exchange**	8	400	136	536
**Forest Fires**	12	450	67	517
**Abalone**	8	2977	1200	4177
**Auto MPG**	8	199	199	398

The experiments are performed and demonstrated using the proposed PSODS trained RBF NN. To prove the efficiency of the proposed optimizer, experiments were also conducted using PSO trained RBF, DS trained RBF and basic RBF. The obtained results are also compared with the results reported in the literature.

### Dataset description

The benchmark dataset are obtained from UCI repository. These seven dataset which are taken into consideration for testing the performance of the proposed PSODS method is described as below:-

- Boston House Price data is concerned about the house price in the area called Boston with 13 attributes such as crime rate, population, pollution level, accessibility to schools, highways, and workplace etc. as inputs and house price as its output. With the total 506 instances, 253 are training instances and 253 instances are used for testing purpose.
- Concrete compressive strength data is having8 input attributes like concrete, water, fly ash, coarse aggregate, fine aggregate etc. and concrete compressive strength as its output. Out of 1030 total samples 680 are used training and 350 samples for testing.
- Air foil self- noise dataset with scaled sound pressure level as its output and 5 other inputs for a total of 1503 samples is also experimented. Where, 1000 instances are trained and 503 instances are tested.
- Istanbul stock exchange dataset deals with the stock exchange returns with 8 other stock exchanges index as its input attributes. For 536 instances, 400 instances are trained and the remaining 136 instances are tested.
- Forest fires dataset is to estimate the burned area of the forest with 12 relevant inputs for estimation and 517 similar instances. Of which 450 are training instances and 67 are testing instances.
- Abalone dataset is used primarily to predict the age of abalone with the help of 8 input attributes and a total of 4177 instances. Out of which 2977 instances as training units and 1200 instances as testing units.
- Auto MPG dataset is mainly used to predict the MPG (miles per gallon) values with the 8 attributes of automobile as its input for 398 samples. 199 samples are used for training and 199 samples are used for testing purpose.

In order to demonstrate the efficacy of the proposed PSODS trained RBF NN, several experiments have been conducted. The PSO and DS also independently used to train the RBF NN for the sake of comparison with the proposed PSODS algorithm. The parameter setting for the DS algorithm is p1 & p2 is set as (0.3xrand), population size is 100. For the PSO algorithm the swarm size is 10 and inertia weight is set = 1. No. of iterations are kept as 1000 for all cases for the PSODS algorithm. The PSO routine will perform the search until 50 iterations or there is no improvement in the solution for 10 iterations.

The results for each dataset using all techniques are obtained by performing the following experiments: Each dataset will be simulated for 30 trial runs to obtain the RMSE having best, worst and mean value. The standard deviation (SD) for each case is also listed. Secondly, to identify the suitable number of hidden neurons for the RBF NN, the hidden neurons is changed in the range of 40 to 70 insteps of 5 neurons and experimented for 30trial runs. In addition, experiments are carried out by varying the training and test samples in contradictory to the standard procedure. Such that, the testing sample are gradually increased by 5% and experimented for 30trial runs using the proposed PSODS trained RBF NN. Error statistics variations are shown to prove the robustness of the PSODS algorithm.

### Performance evaluation on number of hidden neurons

In this section, the RBF NN is trained using the proposed PSODS algorithm along with the DS and PSO algorithms training RBF NN independently. Hidden layer neurons will be fixed in the range of 40 to 70 insteps of 5 neurons and experimented for 30trial runs. All the seven datasets are experimented to decide a most suitable size of hidden layer neurons for effective prediction. The number of training and testing samples are fixed as per the standard figures as given in the UCI database.

**Fig 5:**
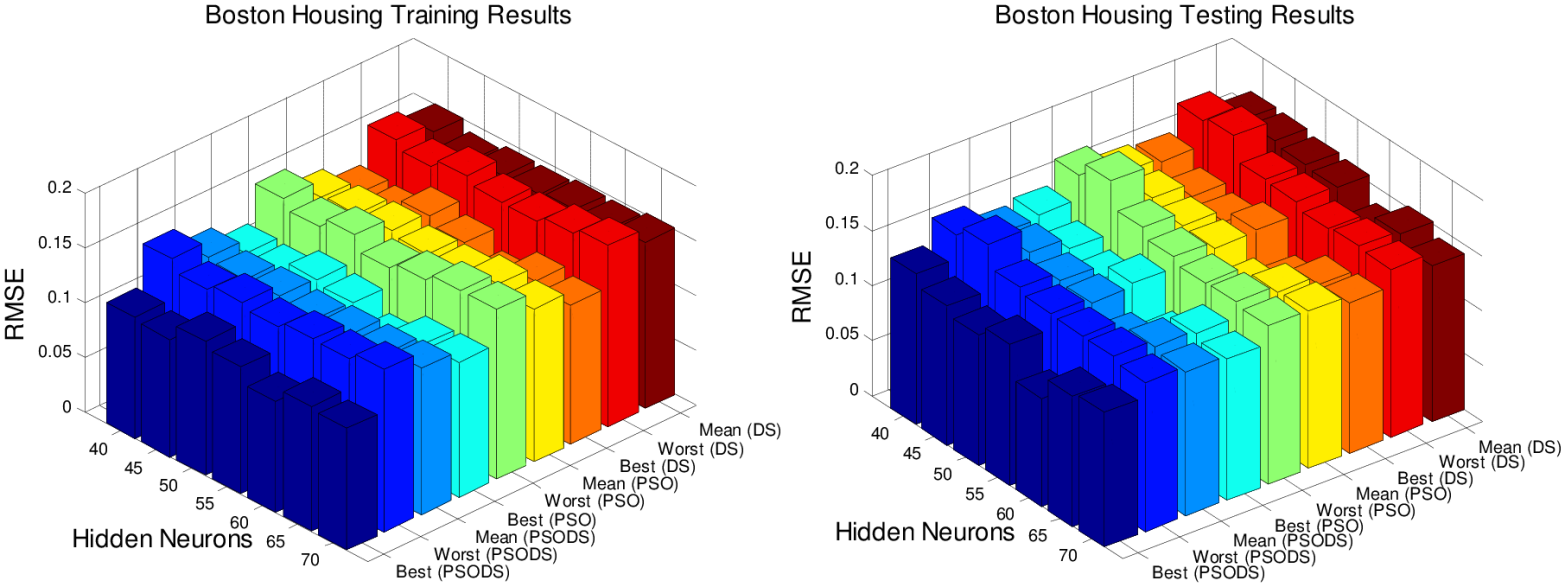
RMSE for Boston House Pricing for by varying hidden layer neurons.

**Fig 6:**
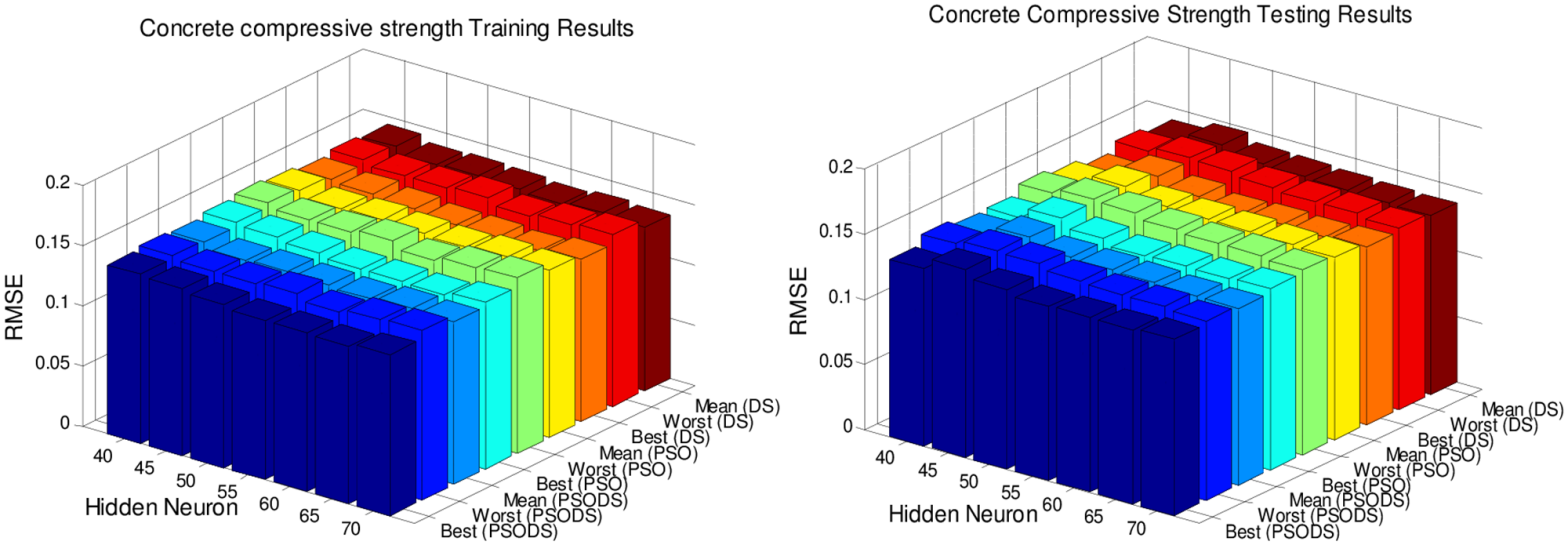
RMSE for Concrete Compressive strength by varying hidden layer neurons.

**Fig 7:**
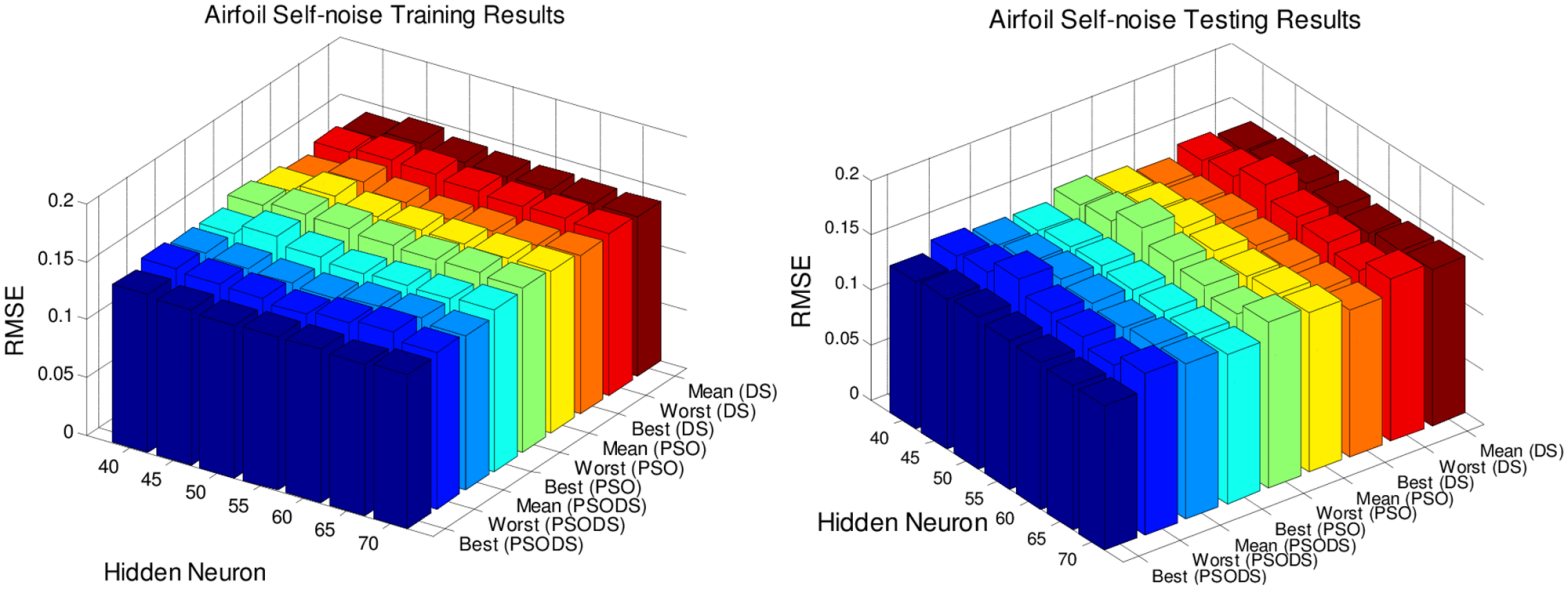
RMSE for Airfoil self—noise by varying hidden layer neurons.

**Fig 8:**
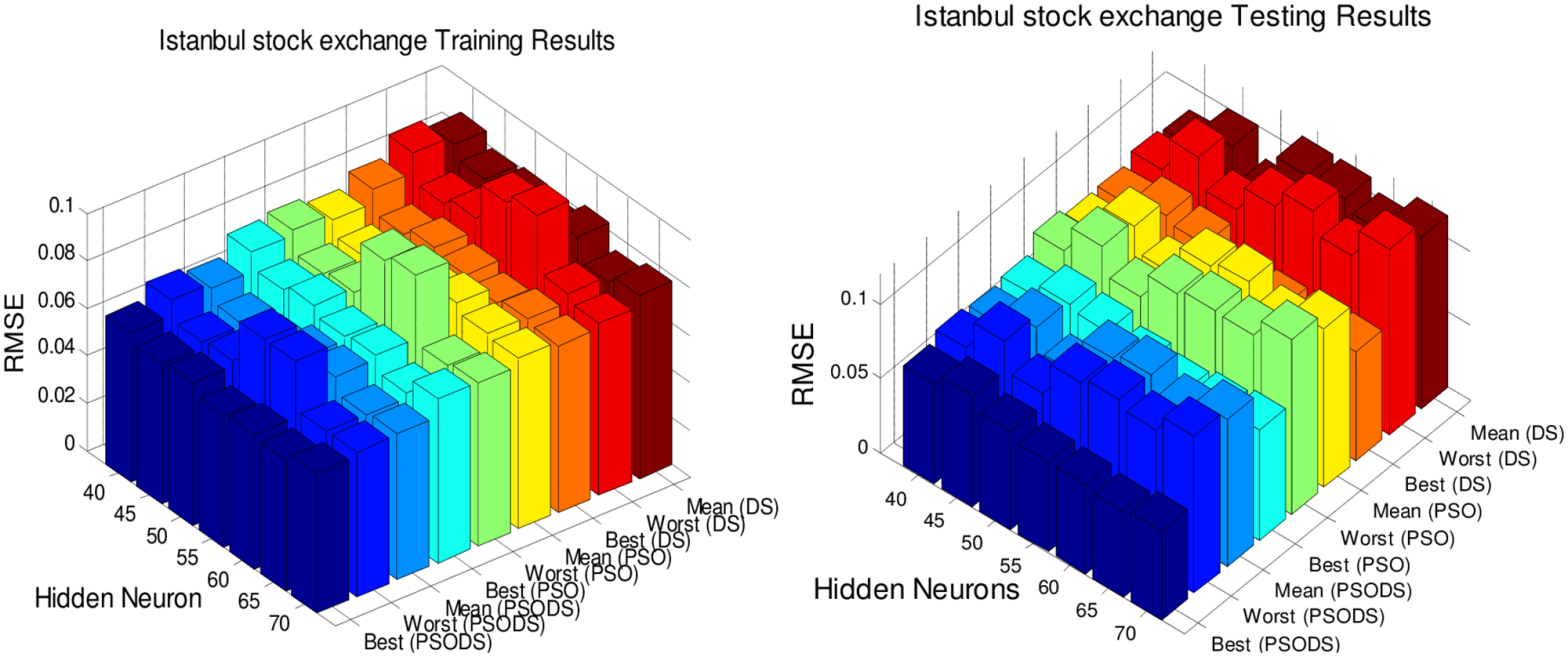
RMSE for Istanbul Stock Exchange by varying hidden layer neurons.

**Fig 9:**
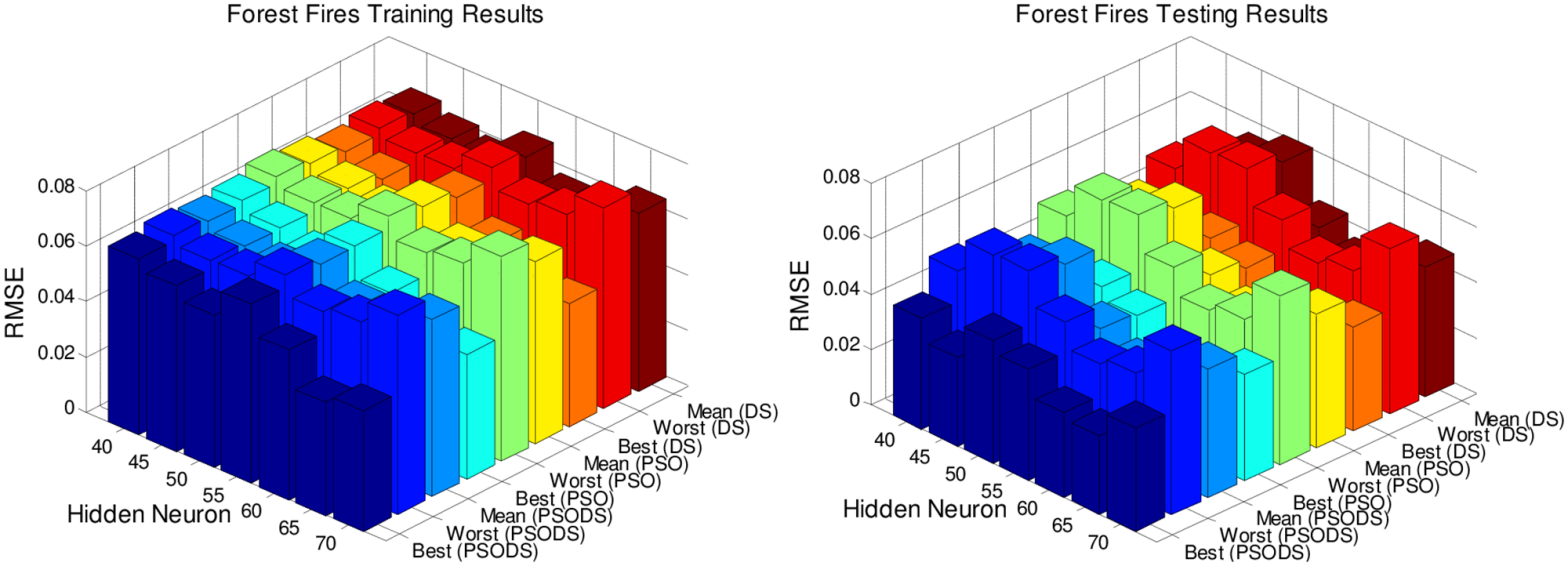
RMSE for Forest Fires by varying hidden layer neurons.

**Fig 10:**
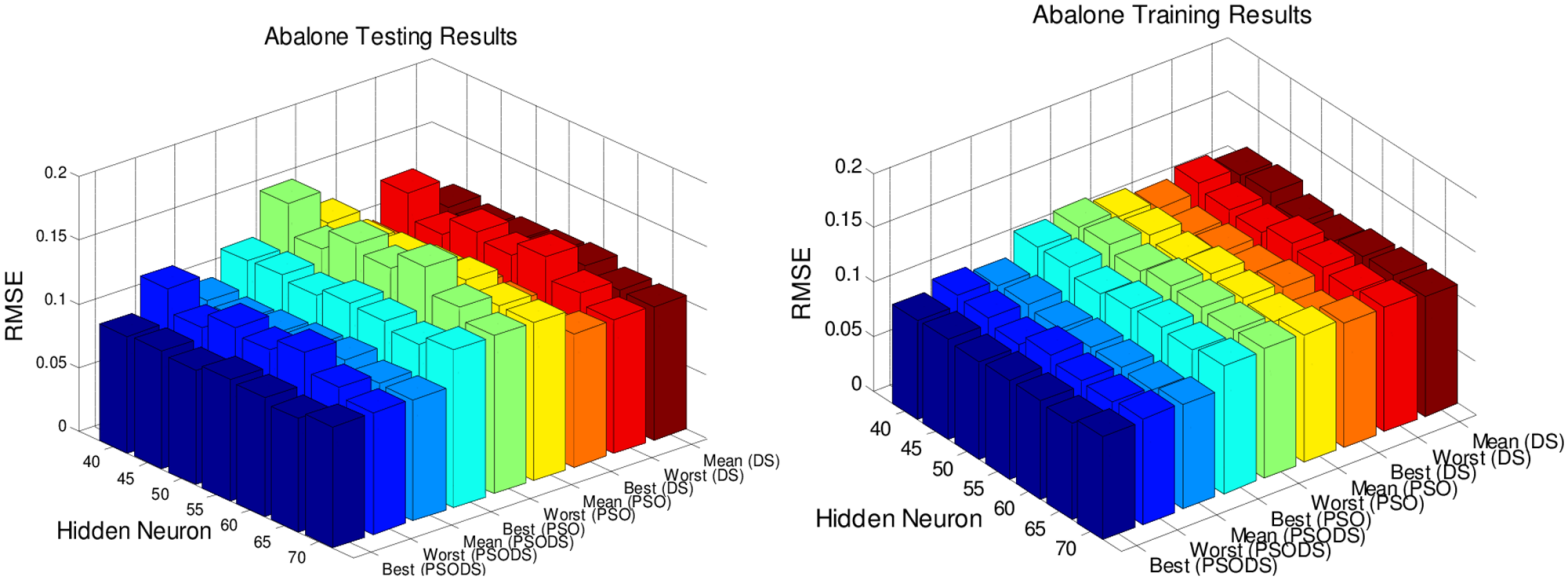
RMSE for Abalone by varying hidden layer neurons.

**Fig 11:**
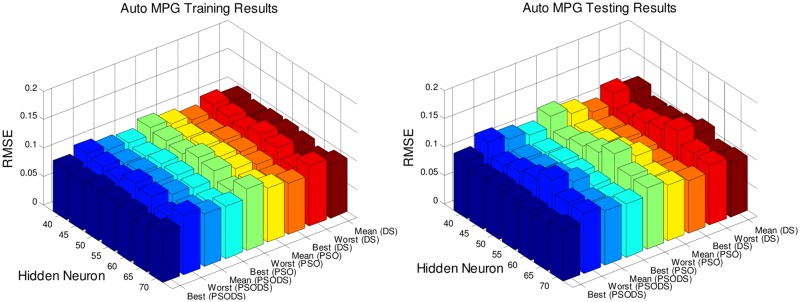
RMSE for Auto MPG by varying hidden layer neurons.

Figs 5 to 11, shows the bar chart for all the seven datasets Boston housing, Concrete Compressive strength, Airfoil self -noise, Istanbul Stock Exchange, Forest Fires, Abalone and Auto MPG respectively. The chart shows the Training RMSE (on left) and Testing RMSE (on right) obtained by the three algorithms.

The following observations can be made from Figs 5 to 11.

As mentioned earlier, the RMSE for both training and testing of samples are plotted against the change in hidden layer neuron size. All the results are based on 30 different trial runs. Since this data is large enough to be tabulated, bar chart in 3D view is plotted. In almost all the datasets the best RMSE is attained at a neuron size of 65.

The PSODS algorithm proves by producing better RMSE compared to the PSO and DS. In some cases the worst results of PSODS are even better than the PSO and DS (e.g., Airfoil self—noise and Abalone). In Boston housing and Istanbul Stock Exchange cases the hidden layer neuron size is close to 60. But still the next better size is 65 and the difference in RMSE produced is also comparatively smaller.

Thus based on the above results, the hidden layer neuron size for the PSODS trained RBF NN is fixed at 65 neurons. Further studies and experiments will be performed with these parameters henceforth of this paper.

Subsequently, the results corresponding to the each dataset used for experimentation are summarized in Tables 2 to 8. Here the RMSE results for proposed PSODS algorithm along with PSO, DS and other algorithms reported in the literature are compared. As mentioned earlier each method will be experimented for 30 trial runs and the tabulated result shows the performance of the algorithms for the RBF NN with 65 neurons in all cases. Here the general RBF NN results are also tabulated as Classic results for the sake of comparison.

**Table 2:** Summary of results obtained for Boston House Pricing.

Dataset	Boston House Pricing		
Method		Train RMSE	Test RMSE
**PSODS**	Best	0.0977	0.1181
	Worst	0.1422	0.1404
	Mean	0.1304	0.1349
	SD	0.0147	0.0094
**PSO**	Best	0.1125	0.1329
	Worst	0.1530	0.1553
	Mean	0.1452	0.1473
	SD	0.0118	0.0075
**DS**	Best	0.1086	0.1297
	Worst	0.1523	0.1506
	Mean	0.1406	0.1468
	SD	0.0121	0.0072
[50,51]	Best	0.0987	0.2413
	Worst	0.1482	0.2621
	Mean	0.1421	0.2589
	SD	0.0154	0.0081
**Classic**	Best	0.1423	0.1681
	Worst	0.1935	0.1964
	Mean	0.1836	0.1863
	SD	0.0149	0.0095

**Table 3:** Summary of results obtained for Concrete Compressive strength.

Dataset	Concrete compressive strength		
Method		Train RMSE	Test RMSE
**PSODS**	Best	0.1269	0.1320
	Worst	0.1410	0.1351
	Mean	0.1346	0.1341
	SD	0.0058	0.0015
**PSO**	Best	0.1386	0.1449
	Worst	0.1521	0.1489
	Mean	0.1459	0.1466
	SD	0.0041	0.0010
**DS**	Best	0.1375	0.1444
	Worst	0.1527	0.1493
	Mean	0.1475	0.1468
	SD	0.0053	0.0013
[50]	Best	0.1453	0.1575
	Worst	0.1652	0.1703
	Mean	0.1573	0.1658
	SD	0.0062	0.0028
**Classic**	Best	0.1516	0.1585
	Worst	0.1664	0.1629
	Mean	0.1596	0.1603
	SD	0.0045	0.0011

**Table 4:** Summary of results obtained for Airfoil self -noise.

Dataset	Airfoil Self-noise		
Method		Train RMSE	Test RMSE
**PSODS**	Best	0.1308	0.1337
	Worst	0.1429	0.1427
	Mean	0.1378	0.1369
	SD	0.0045	0.0032
**PSO**	Best	0.1434	0.1476
	Worst	0.1575	0.1533
	Mean	0.1507	0.1492
	SD	0.0013	0.0028
**DS**	Best	0.1425	0.1445
	Worst	0.1568	0.1543
	Mean	0.1505	0.1477
	SD	0.0027	0.0019
[50,51]	Best	0.1470	0.1373
	Worst	0.1579	0.1724
	Mean	0.1531	0.1593
	SD	0.0072	0.0057
**Classic**	Best	0.1881	0.1936
	Worst	0.2066	0.2011
	Mean	0.1976	0.1957
	SD	0.0017	0.0037

**Table 5:** Summary of results obtained for Istanbul Stock Exchange.

Dataset	Istanbul stock exchange		
Method		Train RMSE	Test RMSE
**PSODS**	Best	0.0552	0.0603
	Worst	0.0806	0.1565
	Mean	0.0663	0.0945
	SD	0.0106	0.0439
**PSO**	Best	0.0665	0.0736
	Worst	0.0941	0.1702
	Mean	0.0785	0.1049
	SD	0.0024	0.0101
**DS**	Best	0.0659	0.0710
	Worst	0.0950	0.1694
	Mean	0.0790	0.1052
	SD	0.0091	0.0375
[50]	Best	0.0996	0.1438
	Worst	0.1213	0.1623
	Mean	0.1179	0.1497
	SD	0.0071	0.0561
**Classic**	Best	0.1129	0.1250
	Worst	0.1598	0.2890
	Mean	0.1333	0.1781
	SD	0.0041	0.0172

**Table 6:** Summary of results obtained for Forest Fires.

Dataset	Forest fires		
Method		Train RMSE	Test RMSE
**PSODS**	Best	0.0408	0.0599
	Worst	0.0636	0.1251
	Mean	0.0522	0.0825
	SD	0.0114	0.0426
**PSO**	Best	0.0539	0.0711
	Worst	0.0762	0.1353
	Mean	0.0626	0.0972
	SD	0.0014	0.0209
**DS**	Best	0.0533	0.0716
	Worst	0.0781	0.1369
	Mean	0.0628	0.0964
	SD	0.0044	0.0166
[51]	Best	0.0831	0.0912
	Worst	0.0972	0.1213
	Mean	0.9312	0.0973
	SD	0.0017	0.0092
**Classic**	Best	0.1007	0.1328
	Worst	0.1423	0.2527
	Mean	0.1169	0.1816
	SD	0.0026	0.0390

**Table 7:** Summary of results obtained for Abalone.

Dataset	Abalone		
Method		Train RMSE	Test RMSE
**PSODS**	Best	0.0884	0.0935
	Worst	0.0939	0.1181
	Mean	0.0912	0.1019
	SD	0.0019	0.0103
**PSO**	Best	0.0987	0.1047
	Worst	0.1057	0.1322
	Mean	0.1013	0.1121
	SD	0.0003	0.0017
**DS**	Best	0.0993	0.1069
	Worst	0.1078	0.1299
	Mean	0.1043	0.1158
	SD	0.0002	0.0008
[52]	Best	2.1100	2.0797
	Worst	0.1457	0.1577
	Mean	0.1373	0.1427
	SD	0.0052	0.0057
**Classic**	Best	0.1284	0.1382
	Worst	0.1393	0.1679
	Mean	0.1348	0.1497
	SD	0.0003	0.0010

**Table 8:** Summary of results obtained for Auto MPG.

Dataset	Auto MPG		
Method		Train RMSE	Test RMSE
**PSODS**	Best	0.0780	0.0844
	Worst	0.0841	0.1065
	Mean	0.0821	0.0926
	SD	0.0025	0.0086
**PSO**	Best	0.0926	0.0983
	Worst	0.0965	0.1187
	Mean	0.0943	0.1042
	SD	0.0013	0.0044
**DS**	Best	0.0906	0.0985
	Worst	0.0981	0.1197
	Mean	0.0940	0.1067
	SD	0.0013	0.0046
[52]	Best	2.7518	2.7968
	Worst	0.1012	0.1179
	Mean	0.9791	0.1091
	SD	0.0071	0.0059
**Classic**	Best	0.1314	0.1395
	Worst	0.1369	0.1684
	Mean	0.1338	0.1478
	SD	0.0018	0.0062

The following observations can be made from Table 2, showing the results for Boston housing. Here, the out of 506 samples 253 samples have been used for training and a same number of samples are used for testing. As can be seen, the proposed PSODS method is superior in terms of producing quality solutions compared to the results of all the other methods tabulated. The PSODS is superior in both training RMSE of 0.0977 and testing RMSE of 0.1181, as in both the cases the RMSE obtained is much less compared to other methods. Followed by the DS method, as its testing RMSE is better compared to other networks [50,51].

**Fig 12:**
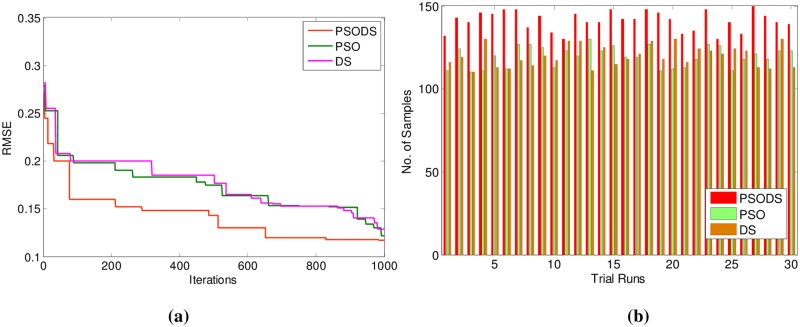
a) Convergence plot b) Successfully predicted samples for Boston House Pricing.

In support of this, Fig 12(a) shows the convergence of the three methods toward best RMSE. As can be seen from the convergence plot, the PSODS algorithm converges faster than the DS and PSO. This plot is one amongst the convergence data in 30 different trial runs. Similarly, Fig 12(b) shows the accuracy in predicting the test sample targets by the RBF trained using three methods. For the sake of leniency of comparison accuracy a tolerance of 0.01 is set for all the methods. Thus the PSODS algorithm has predicted much higher samples (at an average of 147 samples) than the DS (at an average of 125 samples) and PSO (at an average of 110 samples) methods.

Similarly Table 3 shows the results for Concrete Compressive strength. Here, out of 1030 samples 680 samples have been used for training and 350 samples are used for testing. As can be seen, the proposed PSODS method is superior in terms of producing quality solutions compared to the results of all the other methods tabulated. The PSODS is superior in both training RMSE of 0.1269 and testing RMSE of 0.1320, as in both the cases the RMSE obtained is much less compared to other methods, followed by the DS method, as its testing RMSE is better compared to other networks [50].

**Fig 13:**
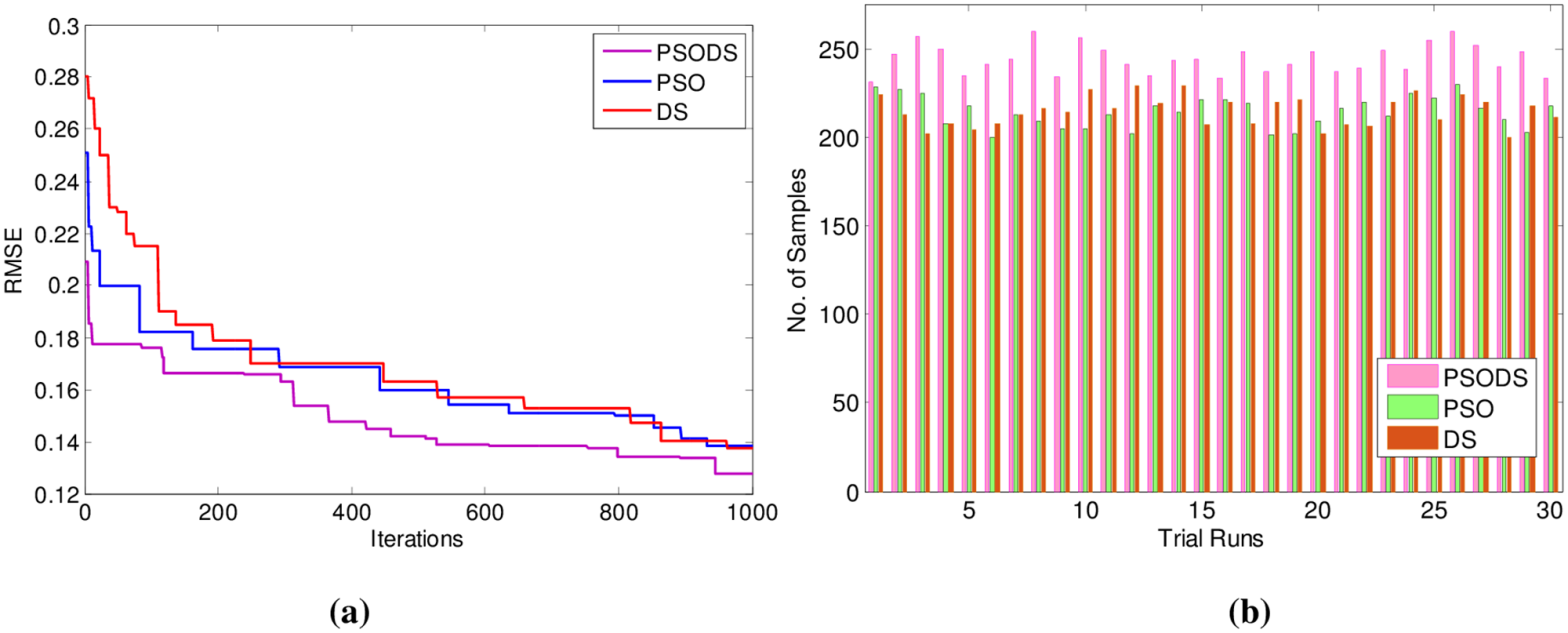
a) Convergence plot b) Successfully predicted samples for Concrete strength.

In support of this, Fig 13(a) shows the convergence of the three methods toward best RMSE FOR Concrete Compressive strength. As can be seen from the convergence plot, the PSODS algorithm converges faster than the DS and PSO. This plot is one amongst the convergence data in 30 different trial runs. Similarly, Fig 13(b) shows the accuracy in predicting the test sample targets by the RBF trained using three methods. For the sake of leniency of comparison accuracy a tolerance of 0.01 is set for all the methods. Thus the PSODS algorithm has predicted much higher samples (at an average of 245 samples) than DS (at an average of 225 samples) and PSO (at an average of 221 samples) methods.

Likewise observations were made from Table 4, showing the results for Airfoil self -noise. Here, out of 1503 samples 1000 samples have been used for training and 503 samples are used for testing. As can be seen, the proposed PSODS method is superior in terms of producing quality solutions compared to the results of all the other methods. The PSODS is superior in both training RMSE of 0.1308 and testing RMSE of 0.1337, as in both the cases the RMSE obtained is much less compared to other methods, followed by the DS method is better compared to others [50,51].

**Fig 14:**
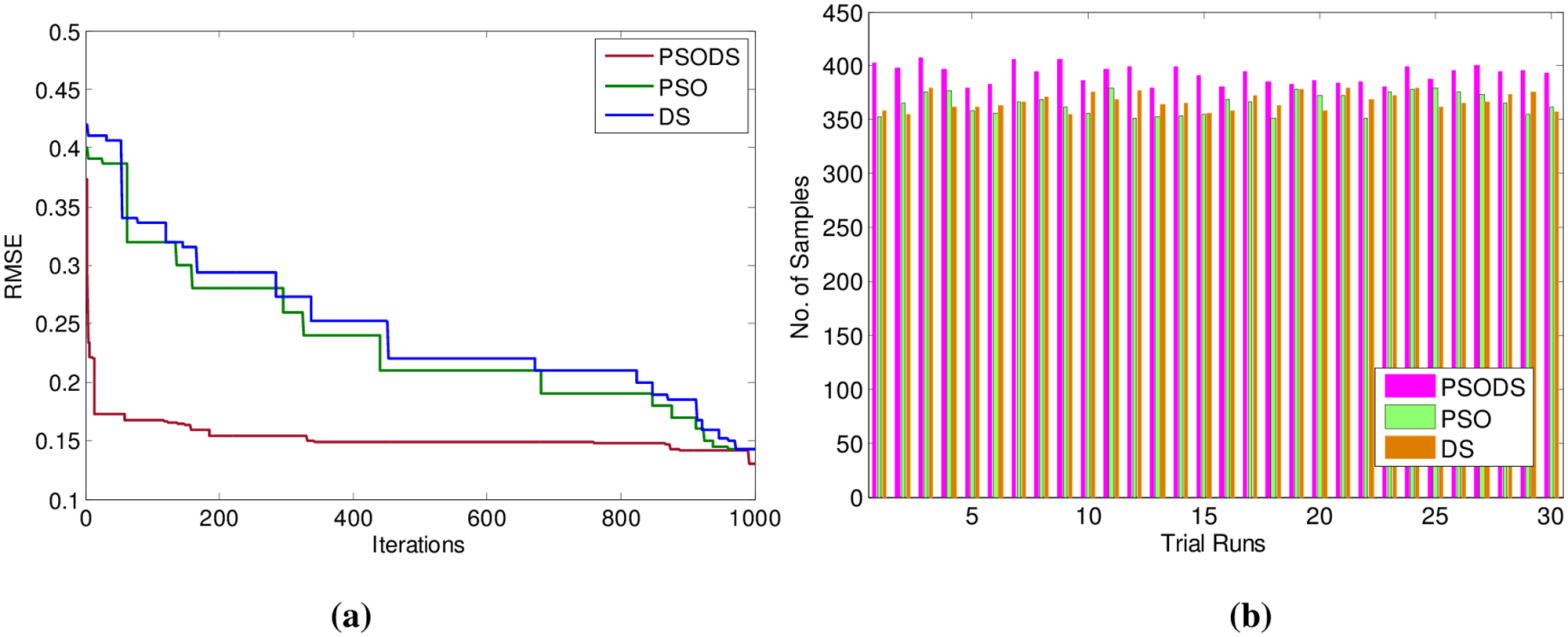
a) Convergence plot b) Successfully predicted samples for Airfoil self -noise.

In support of this, Fig 14(a) shows the convergence of the three methods toward best RMSE for Airfoil self -noise. As can be seen from the convergence plot, the PSODS algorithm converges faster than the DS and PSO. This plot is one amongst the convergence data in 30 different trial runs. Similarly, Fig 14(b) shows the accuracy in predicting the test sample targets by the RBF trained using three methods. For the sake of leniency of comparison accuracy a tolerance of 0.01 is set for all the methods. Thus the PSODS algorithm has predicted much higher samples (at an average of 393 samples) than DS (at an average of 370 samples) and PSO (at an average of 352 samples) methods.

Consequently the results for Istanbul Stock Exchange are shown in Table 5. Here, out of 400 samples 536 samples have been used for training and 136 samples are used for testing. As can be seen, the proposed PSODS method is superior in terms of producing quality solutions compared to the results of all the other methods tabulated. The PSODS is superior in both training RMSE of 0.1269 and testing RMSE of 0.1320, as in both the cases the RMSE obtained is much less compared to other methods, followed by the DS method, as its testing RMSE is better compared to other networks [50].

**Fig 15:**
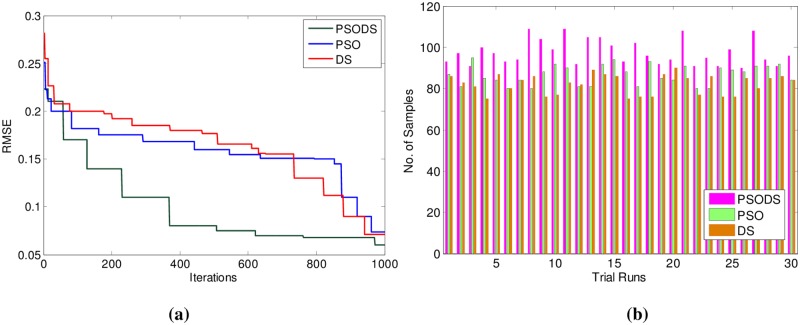
a) Convergence plot b) Successfully predicted samples for Istanbul Stock Exchange.

In support of this, Fig 15(a) shows the convergence of the three methods toward best RMSE for Istanbul Stock Exchange. As can be seen from the convergence plot, the PSODS algorithm converges faster than the DS and PSO. This plot is one amongst the convergence data in 30 different trial runs. Similarly, Fig 15(b) shows the accuracy in predicting the test sample targets by the RBF trained using three methods. For the sake of leniency of comparison accuracy a tolerance of 0.01 is set for all the methods. Thus the PSODS algorithm has predicted much higher samples (at an average of 97 samples) than the DS (at an average of 85 samples) and PSO (at an average of 80 samples) methods.

Next in Table 6 the results for Forest Fires are summarized. Here, out of 517 samples 450 samples have been used for training and 67 samples are used for testing.

As can be seen, the proposed PSODS method is superior in terms of producing quality solutions compared to the results of all the other methods tabulated. The PSODS is superior in both training RMSE of 0.0408 and testing RMSE of 0.0599, as in both the cases the RMSE obtained is much less compared to other methods, followed by the DS method, as its testing RMSE is better compared to other networks [51].

**Fig 16:**
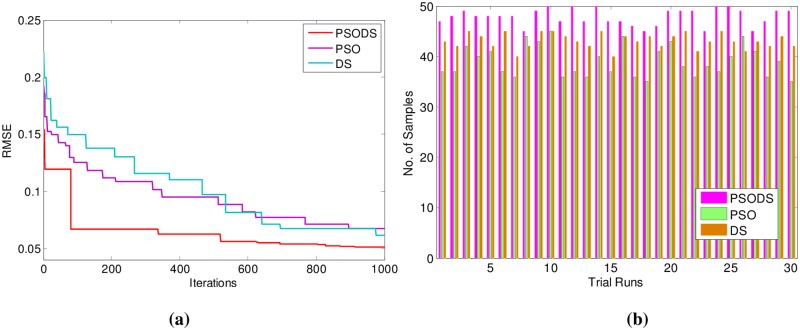
a) Convergence plot b) Successfully predicted samples for Forest Fires.

In support of this, Fig 16(a) shows the convergence of the three methods toward best RMSE for Forest Fires. As can be seen from the convergence plot, the PSODS algorithm converges faster than the DS and PSO. This plot is one amongst the convergence data in 30 different trial runs. Similarly, Fig 16(b) shows the accuracy in predicting the test sample targets by the RBF trained using three methods. For the sake of leniency of comparison accuracy a tolerance of 0.01 is set for all the methods. Thus the PSODS algorithm has predicted much higher samples (at an average of 47 samples) than the DS (at an average of 42 samples) and PSO (at an average of 38 samples) methods.

In continuation following observations were made from Table 7, showing the results for Abalone. Here, out of 4177 samples 2977 samples have been used for training and 1200 samples are used for testing. As can be seen, the proposed PSODS method is superior in terms of producing quality solutions compared to the results of all the other methods tabulated. The PSODS is superior in both training RMSE of 0.0884 and testing RMSE of 0.0935, as in both the cases the RMSE obtained is much less compared to other methods, followed by the DS method, as its testing RMSE is better compared to other networks [51].

**Fig 17:**
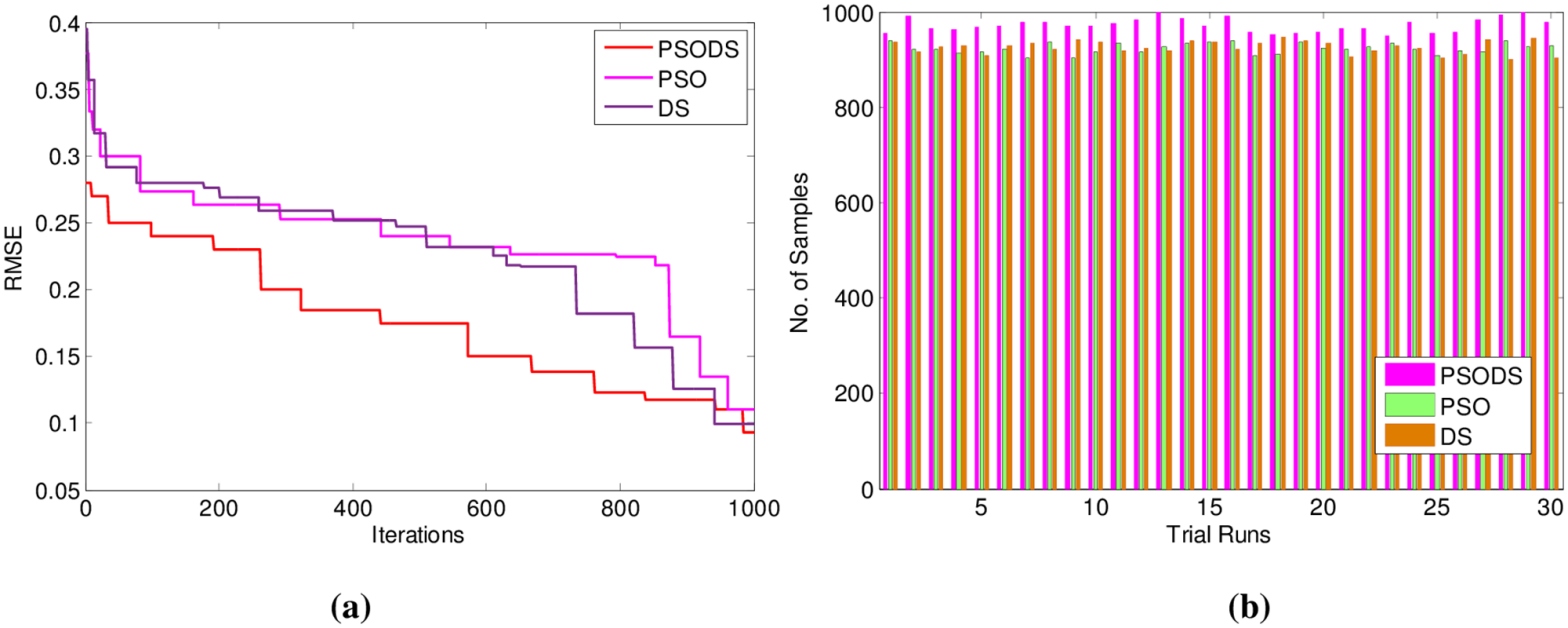
a) Convergence plot b) Successfully predicted samples for Abalone.

In support of this, Fig 17(a) shows the convergence of the three methods toward best RMSE for Abalone. As can be seen from the convergence plot, the PSODS algorithm converges faster than the DS and PSO. This plot is one amongst the convergence data in 30 different trial runs. Similarly, Fig 17(b) shows the accuracy in predicting the test sample targets by the RBF trained using three methods. For the sake of leniency of comparison accuracy a tolerance of 0.01 is set for all the methods. Thus the PSODS algorithm has predicted much higher samples (at an average of 970 samples) than DS (at an average of 925 samples) and PSO (at an average of 910 samples) methods.

Again, the results for Auto MPG are shown in Table 8. Here, out of 398 samples 199 samples have been used for training and 199 samples are used for testing. As can be seen, the proposed PSODS method is superior in terms of producing quality solutions compared to the results of all the other methods tabulated. The PSODS is superior in both training RMSE of 0.0780 and testing RMSE of 0.0844, as in both the cases the RMSE obtained is much less compared to other methods, followed by the DS method, as its testing RMSE is better compared to other networks [52].

**Fig 18:**
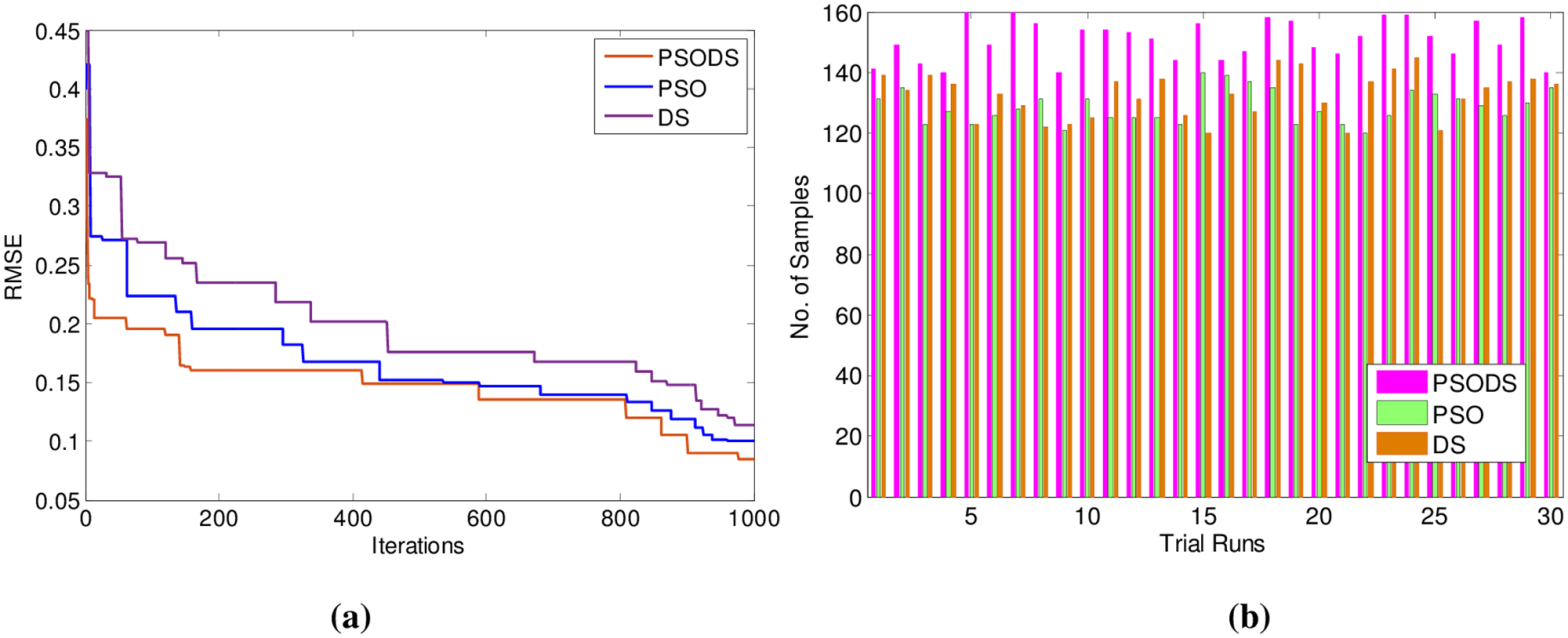
a) Convergence plot b) Successfully predicted samples for Auto MPG.

In support of this, Fig 18(a) shows the convergence of the three methods toward best RMSE for Auto MPG. As can be seen from the convergence plot, the PSODS algorithm converges faster than the DS and PSO. This plot is one amongst the convergence data in 30 different trial runs. Similarly, Fig 18(b) shows the accuracy in predicting the test sample targets by the RBF trained using three methods. For the sake of leniency of comparison accuracy a tolerance of 0.01 is set for all the methods. Thus the PSODS algorithm has predicted much higher samples (at an average of 157 samples) than DS (at an average of 135 samples) and PSO (at an average of 125 samples) methods.

### Comparison of error statistics using PSODS

**Fig 19:**
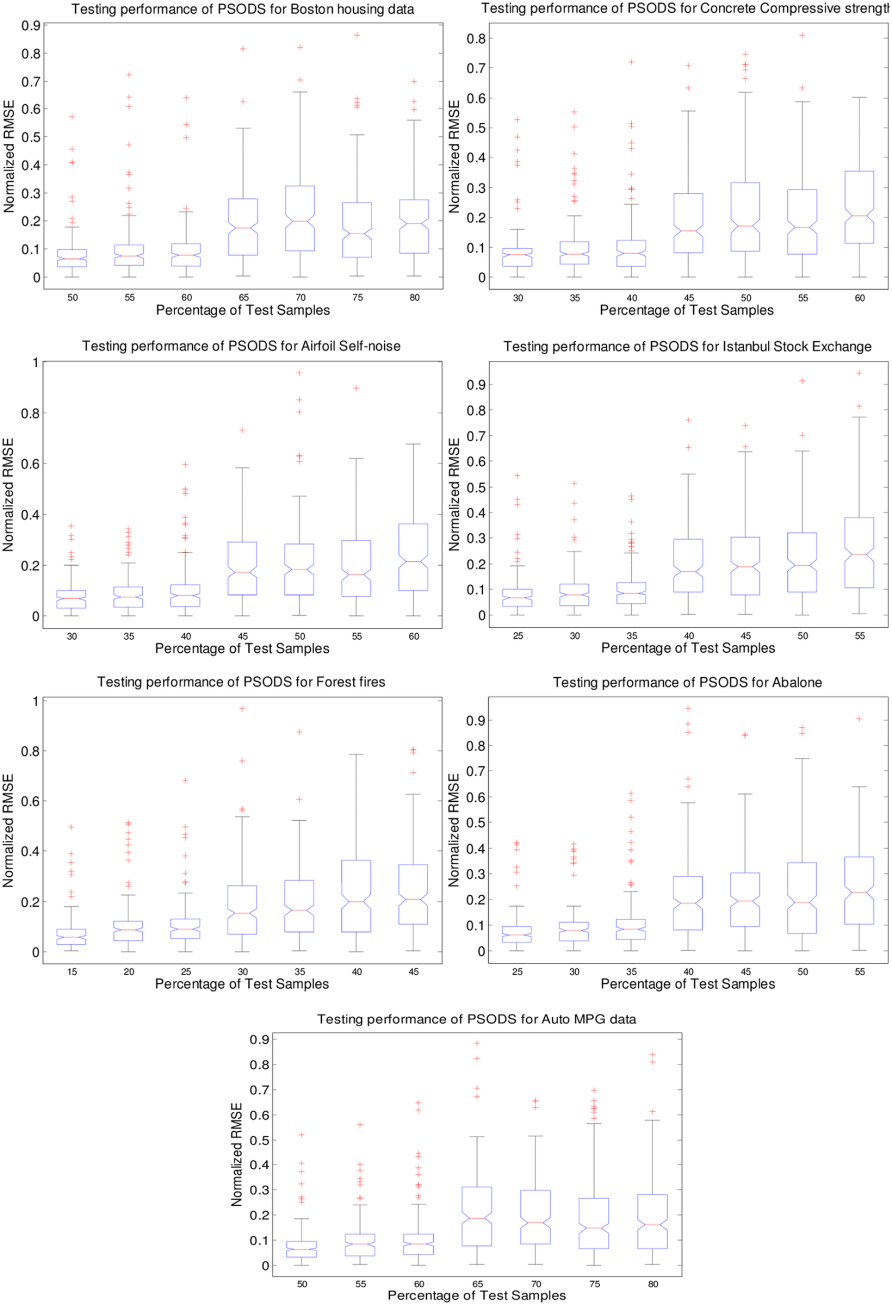
Normalized RMSE statistics using PSODS for % increase in testing samples.

In this experiment, the proposed PSODS trained RBF NN is tested for its applicability in predicting testing sample increased from its standard size. To facilitate this, the training samples are reduced at the rate of 5% from its original size and alternatively the testing samples are equally increased. The seven bench mark datasets are experimented and the box plots are shown in Fig 19. Instead of the RMSE value, the normalized RMSE (NRMSE) value is plotted in order to make easy the comparison between 7 datasets altogether. In order to realize this following Eq (8) is used for calculating the NRMSE:

$$NRMSE=\frac{RMSE}{\bar{L}}$$ (8)

Where, RMSE is the root mean squared error given in (5) and $\bar{L}$ is the difference of the maximum and minimum RMSE of the respective case.

Based on the plots, the following observations are made:

- the error variation is minimum when the testing samples are increased to 10% in almost all cases by the proposed PSODS method. Whereas, when the sample increase goes beyond 15%, the variation is considerably getting higher.
- the median seems to be same for the increase in testing sample for almost 10% and then it also goes little higher for further increase in testing sample.
- the box span indicates the spread of the error and again here up to 10% increase in testing samples the PSODS has produced similar variations.
- the outliers also indicates the performance efficacy in producing similar error statistics for the PSODS method for 10% increase in testing samples (alternatively 10% decrease in training samples)
- perhaps not a full data is presented in this paper about the performance of the PSO and DS methods in this experiment, they could not show any improvement when the test samples are increased.

### Wind speed prediction

This problem is a practical wind prediction problem and its data is measured by Suzlon Energy Ltd, India, during June 2015. The terrain is tropical (Palghat Pass, India) and data is regressive. The data description is similar to other wind prediction models. Thus, the wind speed as desired output and its corresponding atmospheric variables such as wind vane direction, temperature, atmospheric pressure, air density and relative humidity for the altitude of 65m as input attributes are obtained from Suzlon Energy Ltd, India. The total of 832 hourly data samples are considered, of which 500 samples are used for training and 332 samples are used for testing the performance of the algorithm.

Based on the performance of the RBF NN trained by PSODS algorithm, the hidden layer neuron is set to 65. The simulation parameters are set as it is for both the algorithms while solving the 7 benchmark datasets. Simulation for wind prediction is done for 30trial runs using three algorithms to train the RBF NN. Table 9 summarizes the results obtained and depicts the superiority of the proposed PSODS algorithm over PSO and DS in all cases.

**Table 9:** Summary of results obtained for wind speed.

	Wind Speed		
Method		Train RMSE	Test RMSE
**PSODS**	Best	0.181989	0.195412
	Worst	0.196687	0.200098
	Mean	0.189058	0.198033
	SD	0.006013	0.001936
**PSO**	Best	0.190602	0.184147
	Worst	0.199691	0.200109
	Mean	0.195708	0.191417
	SD	0.003794	0.006675
**DS**	Best	0.188173	0.188101
	Worst	0.195178	0.203312
	Mean	0.191780	0.195275
	SD	0.003157	0.005547

**Fig 20:**
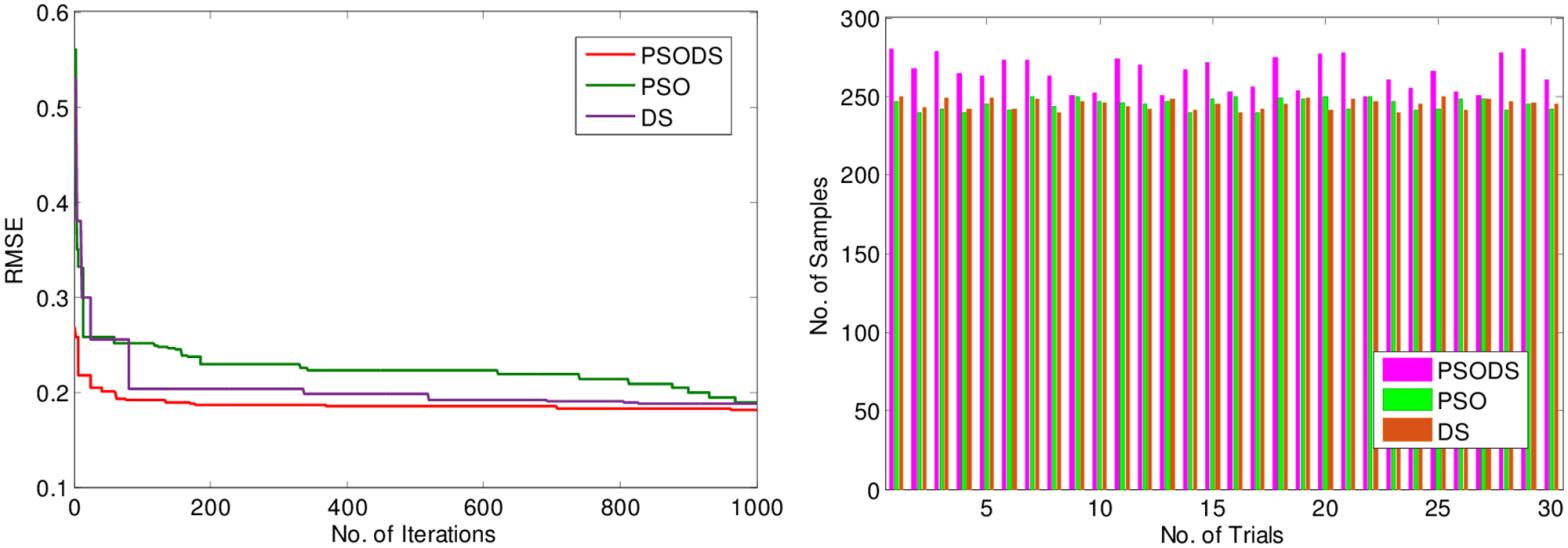
a) Convergence plot b) Successfully predicted samples for wind speed.

Similarly, the convergence plot for 1000 iterations is shown in Fig 20(a). Again the proposed PSODS algorithm outperforms the other two algorithms and reaches the better solution faster. Due to the search feature blended from both DS and PSO algorithms, the PSODS reaches the quality solution in early iterations itself. In the same way, Fig 20(b), depicts the plot of three algorithms in attaining the accuracy by predicting the target samples. According the plot elucidates the successful numbers of samples predicted with a 0.01 tolerance. Based on this plot, it is comprehensible that the PSODS algorithm could make the RBF NN to predict relatively higher number of samples, compared to the PSO and DS methods.

**Fig 21:**
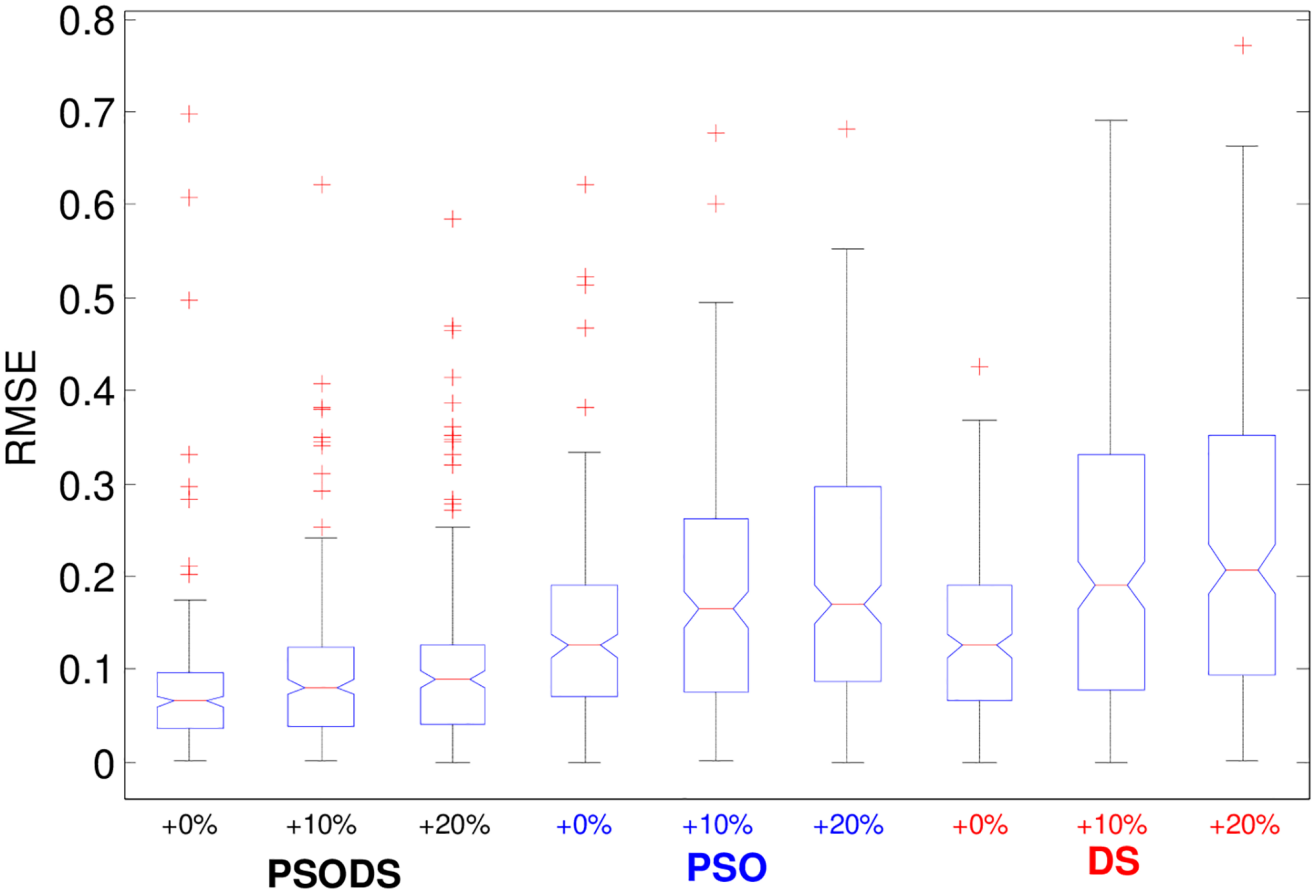
Normalized RMSE statistics for wind speed for % increase in testing samples.

To additionally substantiate the performance of the proposed PSODS algorithm over PSO and DS methods, Fig 21, portray the variance plot of the three methods to obtain the RMSE for increase in testing samples. Here each algorithm is used to obtain the RMSE for 30 trial runs. As discussed earlier, there are 832 hourly data samples, of which 500 samples are used for training and 332 (+0%) samples are used for testing. While doing the simulations, the testing samples are increased to 10% and 20%. Accordingly the Training sample will be reduced. Intelligibly from Fig 21, the PSODS algorithm could predict better results compared to the other two methods. Thus again PSODS establishes itself as a suitable method for prediction.

To demonstrate the superiority of the proposed PSODS technique over the other existing neural network method for wind speed prediction, three networks are chosen and experimented for 30 trial runs. The three NN are basic RBF NN [19], extreme learning machine (ELM) [50] and multi-layer perceptron [16] trained by back propagation algorithm (MLP-BP). Table 10, summarizes the test RMSE obtained by various methods for predicting the datasets and wind speed. During the experiments the training samples are kept at 40% and 60% of samples are taken for testing. From the table, it can be observed that the proposed PSODS trained RBF NN is dominant in predicting the samples compared to other methods.

**Table 10:** Summary of test RMSE obtained using different neural networks.

Datasets	Test RMSE			
	PSODS-RBFNN	RBFNN	ELM	MLP-BP
**Boston housing**	0.1181	0.1681	0.1202	0.1924
**Concrete Compressive strength**	0.1320	0.1585	0.1337	0.1912
**Airfoil self -noise**	0.1337	0.1936	0.1342	0.2152
**Istanbul Stock Exchange**	0.0603	0.1250	0.0651	0.1562
**Forest Fires**	0.0599	0.1328	0.0621	0.1717
**Abalone**	0.0935	0.1382	0.0945	0.1882
**Auto MPG**	0.0844	0.1395	0.0861	0.1628
**Wind Speed**	0.1954	0.2105	0.1983	0.2514

Table 11, summarizes the accuracy in predicting the test sample targets by the three networks. For the sake of leniency of comparison accuracy a tolerance of 0.01 is set for all the methods. It is observed that the proposed PSODS trained RBF is superior in terms of producing successful samples compared to the results of all the other networks tabulated. Thus the PSODS trained RBF is superior in predicting for all the 7 datasets and the wind problem with less testing RMSE and also by predicting more number of successful test samples when compared to other NN for a 30 trial experiment.

**Table 11:** Summary of successful prediction of samples.

Datasets	Successful test samples predicted			
	PSODS-RBFNN	RBFNN	ELM	MLP-BP
**Boston housing**	147	135	130	120
**Concrete Compressive strength**	245	210	227	204
**Airfoil self -noise**	393	362	371	347
**Istanbul Stock Exchange**	97	81	86	72
**Forest Fires**	47	37	40	32
**Abalone**	970	951	945	903
**Auto MPG**	157	133	146	127
**Wind Speed**	300	283	291	278

Similarly, in order to justify the merits of the proposed chaotic opposition-based population (COP) initialization algorithm over the general population (GP) initialization algorithm is experimented to show the dominance of the former in supporting the PSODS trainer to swiftly predict the test sample. Accordingly, experiments are conducted for 30 trials and Table 12, summarizes the results obtained. For this purpose, the best and worst fitness value (RMSE) is recorded during initialization along with the final solution obtained by the PSODS-RBF NN on termination of the algorithm.

**Table 12:** Performance comparison of population initialization algorithm for PSODS-RBFNN.

Datasets	RMSE					
	Initial Best COP	Initial Best GP	Initial worst COP	Initial worst GP	Best using COP	Best using GP
**Boston housing**	0.3241	0.5211	0.3715	0.7174	0.1181	0.1914
**Concrete Compressive strength**	0.4007	0.6104	0.4275	0.8145	0.1320	0.1821
**Airfoil self -noise**	0.3710	0.5112	0.4003	0.7721	0.1337	0.2147
**Istanbul Stock Exchange**	0.1121	0.3371	0.1745	0.5141	0.0603	0.1507
**Forest Fires**	0.1054	0.3020	0.1257	0.5871	0.0599	0.1625
**Abalone**	0.2471	0.4014	0.3011	0.7419	0.0935	0.1719
**Auto MPG**	0.2661	0.5071	0.2914	0.8019	0.0844	0.1421
**Wind Speed**	0.3877	0.5412	0.4107	0.7721	0.1954	0.2376

From the summarized results it is clear that, the proposed chaotic opposition-based population initialization algorithm could generate better initial search solutions and reach the better solution region and produce quality RMSE over the regular random initialization algorithm. Also the PSODS-RBFNN with COP could reach the better solution well before the termination criterion. Thus the proposed chaotic opposition-based population initialization algorithm has great influence of the training and convergence of the proposed PSODS algorithm.

## Conclusions

This paper presents an integrated hybrid optimization algorithm for training the radial basis function neural network for prediction of standard benchmark regression data sets and one real-time wind speed case. Accordingly, a hybrid training procedure with differential search DS algorithm functionally integrated with the PSO is modelled and experimented. Here the DS will be used as the main optimizer and PSO will use a neighbourhood topology to exploit the solutions of DS by thorough search of solution region. This neighbourhood topology will be based on ring topology with neighbours fetched considering both fitness and candidates themselves. A new chaotic map based algorithm to generate the initial population is proposed to support the PSODS algorithm to search the n-dimensional space thoroughly by supplementing the diversity of population and reach better optimum regions swiftly. To exemplify the potency of the PSODS method and to generalize the RBF NN architecture, scrupulous experiments are carried out to find the optimum size of hidden layer neurons.

The Numerical experiments on publicly available 7 benchmark datasets are performed using the proposed PSODS algorithm for 30 trial runs to evaluate the RMSE to ensure the RBF NN is prepared to predict outputs of regressive samples database. In all cases the PSODS outperforms the PSO and DS methods in terms of convergence rate and is reliable as the error statics variations are fairly petite. To demonstrate the applicability of the PSODS with reduced samples for training, experiments are carried out by reducing training samples and tested with increased samples, again the error statistics proves the PSODS method is robust in prediction. The prediction accuracy is also demonstrated by evaluating the number of samples closely (0.01 tolerance) predicted by all the three methods for training RBF NN. Also a standard of 1000 iterations is fixed for all the three methods. Subsequently, experiments were conducted on a practical application case for wind speed prediction to expound the superiority of the proposed PSODS training algorithm in terms of prediction accuracy.

In extended work, the proposed PSODS method to train RBF NN will be demonstrated for problems with more attributes and problems with missing data. Also simulations with other types of neural networks such as Extreme learning machines (ELM) will be significant. Also, it is worth to further navigate the proposed PSODS algorithm with many prediction problems such as electricity price forecasting, solar irradiance and solar radiation prediction.
